# Targeting G protein-coupled receptor signaling at the G protein level with a selective nanobody inhibitor

**DOI:** 10.1038/s41467-018-04432-0

**Published:** 2018-05-18

**Authors:** Sahil Gulati, Hui Jin, Ikuo Masuho, Tivadar Orban, Yuan Cai, Els Pardon, Kirill A. Martemyanov, Philip D. Kiser, Phoebe L. Stewart, Christopher P. Ford, Jan Steyaert, Krzysztof Palczewski

**Affiliations:** 1 Department of Pharmacology, School of Medicine, Case Western Reserve University, 10900 Euclid Avenue, Cleveland, OH 44106 USA; 2 Cleveland Center for Membrane and Structural Biology, Case Western Reserve University, 1819 East 101st Street, Cleveland, OH 44106 USA; 3 Department of Neuroscience, The Scripps Research Institute, 130 Scripps Way, Jupiter, FL 33458 USA; 4 Department of Physiology and Biophysics, School of Medicine, Case Western Reserve University, 10900 Euclid Ave, Cleveland, OH 44106 USA; 5 Department of Pharmacology, University of Colorado School of Medicine, 12800 East 19th Ave, Aurora, CO 80045 USA; 6 Structural Biology Brussels, Vrije Universiteit Brussel (VUB), Brussels, 1050 Belgium; 7 VIB-VUB Center for Structural Biology, VIB, Brussels, 1050 Belgium; 8 Research Service, Louis Stokes Cleveland VA Medical Center, Cleveland, OH 44106 USA

## Abstract

G protein-coupled receptors (GPCRs) activate heterotrimeric G proteins by mediating a GDP to GTP exchange in the Gα subunit. This leads to dissociation of the heterotrimer into Gα-GTP and Gβγ dimer. The Gα-GTP and Gβγ dimer each regulate a variety of downstream pathways to control various aspects of human physiology. Dysregulated Gβγ-signaling is a central element of various neurological and cancer-related anomalies. However, Gβγ also serves as a negative regulator of Gα that is essential for G protein inactivation, and thus has the potential for numerous side effects when targeted therapeutically. Here we report a llama-derived nanobody (Nb5) that binds tightly to the Gβγ dimer. Nb5 responds to all combinations of β-subtypes and γ-subtypes and competes with other Gβγ-regulatory proteins for a common binding site on the Gβγ dimer. Despite its inhibitory effect on Gβγ-mediated signaling, Nb5 has no effect on Gα_q_-mediated and Gα_s_-mediated signaling events in living cells.

## Abstract (web summary)

G protein-coupled receptors (GPCRs) activate and dissociate the G protein heterotrimer into Gα-GTP and Gβγ dimer, which facilitate distinct signalling events. Here authors develop a nanobody, Nb5 that modulates Gβγ-mediated signaling without affecting GTP-bound Gαq and Gαs-mediated signaling events.

## Introduction

G protein-coupled receptors (GPCRs) function by translating extracellular stimuli across the plasma membrane into intracellular signaling events^1^. The latter are accomplished by a ligand-induced conformational change in the GPCR that activates a downstream heterotrimeric G protein^2^. Heterotrimeric G proteins, consisting of three subunits, Gα, Gβ and Gγ, undergo a Gα-GDP/GTP exchange that leads to dissociation of the Gβγ dimer from the heterotrimer^3^. Both these moieties then become free to act upon their downstream effector molecules and thereby initiate a variety of intracellular signaling events that constitute the overall GPCR-mediated response. Notably, heterotrimeric G proteins maintain an equilibrium between their heterotrimeric and dissociated states by undergoing spontaneous GPCR-independent association/dissociation^4^.

While GPCRs serve as the largest class of drug-targeted membrane proteins, producing the majority of FDA-approved drugs available on the market, only a handful of GPCR-mediated signaling events have been targeted therapeutically with either small molecule or peptide modulators^5^. Such modulators can be classified as either agonists, antagonists, or inverse agonists, depending on their ability to either stabilize the activated state of receptors, inhibit agonist competitively, or reduce the basal spontaneous coupling to G proteins, respectively^6^. Some biased signaling ligands stabilize an intermediate receptor conformation that mimics the active conformation with respect to one signaling pathway, while simultaneously mimicking the inactive conformation for another signaling pathway^7^. However, drug development for GPCR signaling pathways has been hampered by difficulties in identifying molecules with suitable selectivity^8^. Related GPCRs share a promiscuous ligand-binding site for lipophilic ligands, which allows several off-target effects when pursued therapeutically. Additionally, lipophilic ligands can penetrate the central nervous system (CNS), and thereby interact with undesirable neuronal receptor targets. As an alternative strategy, functional monoclonal antibodies (mAbs) are currently being used to target less well-conserved allosteric sites of GPCRs to selectively modulate their signaling^9^. Although mAbs and their fragments have played an instrumental role in selectively targeting GPCR signaling pathways, their commercial success is limited due to their high costs of production and treatment, especially when their efficacy in prolonging survival is similar to that of less expensive alternatives.

Antibody alternatives are known to be versatile tools for exploring the determinants of GPCR recognition. However, nanobodies (Nbs) derived from the variable region of camelid heavy chain are endowed with favorable characteristics in terms of size, solubility, affinity, and ease of production^10^. Nbs are single-domain entities which form the smallest antigen binding fragment that completely retains the binding affinity and specificity of a full-length antibody^10^. Nbs possess exceptionally long complementarity-determining region 3 (CDR3) loops and a convex paratope, which allow them to penetrate into hidden cavities of target antigens^11^. These unique biochemical and biophysical properties of Nbs render them superior to conventional mAbs or antibody fragments, and ideally suit them for a myriad of biotechnological applications. In addition to their use in GPCR structural studies, therapeutic Nbs are being discovered that target GPCR signaling^12^. A biparatopic nanobody targeting different binding sites on the chemokine receptor CXCR2 recently entered phase I studies for therapy of inflammation^13^. Additionally, Nbs targeting chemokine receptor CXCR4 were found to inhibit chemotaxis and HIV-1 replication in vivo^12^. vNARs^14^, the variable domain of shark-derived new antigen receptors and their human equivalents, i-bodies^15^ derived from the I-set immunoglobulin superfamily are other examples of single-domain antibody-like molecules with biochemical and biophysical properties similar to Nbs. Recently, i-bodies that selectively block CXCR4 β-arrestin recruitment were reported^15^. With the exception of β-arrestin specific antibody fragments^16^, all small-molecule-based, antibody-based, and nanobody-based approaches developed to date target GPCR-mediated signaling at the GPCR level^12^, and thereby are GPCR-specific and cannot be used universally. Additionally, these approaches activate both Gα-GTP-mediated and Gβγ-mediated signaling pathways that lead to the activation of undesired cellular signaling events.

The liberated Gβγ dimer is a very efficient signal transducer^17–21^ and can dysregulate various cellular functions leading to numerous side effects associated with dysregulated ion channels, phosphoinositide-3-kinase (PI3K), adenylyl cyclase (AC) and mitogen-activated protein kinase (MAPK) pathways. These diverse Gβγ functions make it an attractive target for the treatment of many medical conditions^22^. However, the ability of Gβγ to play essential roles in various cellular functions, including the formation of heterotrimeric G proteins, necessitates highly-specific Gβγ inhibitors that modulate its function while keeping Gα-mediated signaling intact. Here we describe a nanobody (Nb5) that specifically binds to the Gβγ dimer and shifts the association/dissociation equilibrium of the heterotrimeric visual G protein (G_t_) towards dissociated Gα_t_ and Nb5-bound Gβ_1_γ_1_ subunits. Differential hydrogen/deuterium exchange (HDX) and crystallography studies suggest a competition between Nb5 and other Gβγ-regulatory proteins for a common binding site on the Gβγ dimer. Interestingly, Nb5 binds and inhibits Gβ subtypes 1–4, showing its broad applicability to various cell types. Whole-cell patch clamping of striatum neurons demonstrates an inhibitory effect of Nb5 on the activation of Gβγ-regulated G-protein-gated inward rectifier potassium (GIRK) channels. Finally, Nb5 acts as a control-switch of GPCR-mediated cellular signaling by modulating PI3K-protein kinase B/AKT and MAPK-extracellular signal-regulated kinase (ERK) pathways. However, Nb5 does not alter Gα_i_-GTP-governed cellular levels of secondary messenger cyclic adenosine monophosphate (cAMP). Additionally, Nb5 shows no effect on either Gα_q_-induced intracellular Ca^2+^ elevation or Gα_s_-induced cAMP production. This is the first demonstration of a selective inhibitory effect of a nanobody on Gβγ-mediated signaling events that does not affect Gα signaling on a cellular level. While serving as a rare example wherein a nanobody has an inhibitory role, this study also opens potential avenues for nanobody-mediated Gβγ-signaling modulation to treat various excitatory neurological conditions and block cancer progression.

## Results

### Generation of Nbs directed towards Gβ_1_γ_1_

A total of 17 different nanobody clones were identified after ELISA selection wherein the wells were coated with purified bovine Gβ_1_γ_1_ dimer. Nbs were divided into 14 families based on their amino acid sequence. All Nbs were produced as soluble His-tagged protein products in the *E. coli* periplasmic region. Initial binding analyses performed with an immobilized-Ni^2+^ affinity chromatography pull down assay identified 3 Nbs that bound the Gβ_1_γ_1_ dimer. Interestingly, all 3 Gβ_1_γ_1_-positive Nbs belonged to the same nanobody family with an identical complementarity determining region 3 (CDR3) and displayed similar biochemical properties (Supplementary Figure 1). The nanobody with highest expression levels, Nb5, was used for further characterization. Additionally, an irrelevant nanobody, Nb17 was chosen as a negative control due to its non-reactivity with bovine rod outer segments (ROS) proteins.

### Nb5 shifts the dynamic equilibrium of heterotrimeric G_t_

**Fig. 1:**
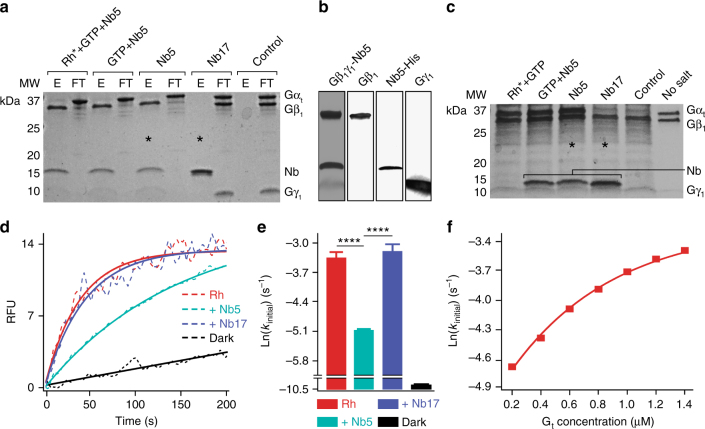
Nanobody-mediated shift in heterotrimeric G_t_ equilibrium. **a** Effect of Nb5 on the heterotrimeric state of G_t_ in solution. Lane 1 shows the dissociation of heterotrimeric G_t_ in the presence of light-activated Rh (Rh*), GTP, and Nb5. Gβ_1_γ_1_ was later co-purified with Nb5 by immobilized-Ni^2+^ affinity chromatography and Gα_t_ emerged as an unbound protein in the flow-through (lane 2). Note the decrease in Gγ_1_ Coomassie staining in the presence of 300 mM imidazole during protein elution. Similar results were obtained from the reaction mixture in the absence of either Rh* alone (lanes 3 and 4) or both Rh* and GTP (lanes 5 and 6). However, Nb17 did not affect the heterotrimeric configuration of G_t_ (lanes 7 and 8, compare lanes marked with asterisks). Additionally, non-specific interactions between Gβ_1_γ_1_ and Ni^2+^-NTA resin were not seen (lanes 9 and 10). The eluate and flow-through obtained from affinity chromatography purification are denoted as E and FT, respectively. **b** Proteins in typical eluates obtained after immobilized-Ni^2+^ affinity chromatography were analyzed by SDS-PAGE (lane 1) and immunoblotting with antibodies specific to Gβ_1_, His-tag, and Gγ_1_, respectively (lanes 2–4). **c** Light activation of ROS membranes in the presence of GTP released Gα_t_ in the high salt wash (lane 1). Similar Gα_t_ release was obtained with Nb5 with or without GTP during the high salt wash (lanes 2 and 3). Treatment of ROS membranes with Nb17 had no effect on Gα_t_ release (lane 4, compare lanes marked with asterisks). Untreated ROS membranes showed no Gα_t_ release during the high salt wash (lane 5). **d** The initial G_t_ activation rate of Rh* was reduced upon pre-treatment of heterotrimeric G_t_ with Nb5 (greencyan) as compared to either untreated (red) or Nb17-treated G_t_ (purple). **e** Quantification of the initial G_t_ activation rates of Rh* with and without nanobody pre-treatment. Results are expressed as the mean ± SD, *n* = 3 replicates, ****P < 0.0001, Student’s *t*-test. **f** The decrease in G_t_ activation rates after Nb5 treatment were explained by Rh single turnover experiments that exhibited an exponential increase in G_t_ activation rates with increasing concentrations of G_t_

The effect of Nb5 on the heterotrimeric state equilibrium of G_t_ was determined by assessing its ability to bind and pull out the β_1_γ_1_ subunits of G_t_ (Gβ_1_γ_1_) from no-salt extracts of ROS. No-salt extracts were chosen over purified G_t_ to ensure its heterotrimeric configuration as the purification procedure could cause partial dissociation of G_t_ subunits. As expected, complete G_t_ heterotrimer dissociation was observed in the presence of light-activated rhodopsin (Rh*) and GTP. As a result, Nb5 bound to the released Gβ_1_γ_1_ dimer and the Gβ_1_γ_1_-Nb5 complex was obtained in the eluate upon immobilized-Ni^2+^ affinity chromatography purification (Fig. 1a, lane 1 and Fig. 1b). In contrast, the displaced alpha subunit of G_t_ (Gα_t_) was found in the flow-through (Fig. 1a, lane 2). Interestingly, similar results were obtained in the absence of either Rh* or Rh* and GTP during the purification (Fig. 1a, lanes 3–6). Moreover, an irrelevant nanobody, Nb17 failed to cause dissociation of the G_t_ heterotrimer (Fig. 1a, compare lanes marked with asterisks). Additionally, no non-specific interactions were seen between Gβ_1_γ_1_ and the Ni^2+^-NTA purification resin (Fig. 1a, lanes 9 and 10). These experiments indicate an Nb5-mediated shift in the equilibrium of heterotrimeric G_t_ in solution towards its dissociated subunits.

Next, the effect of Nb5 on the heterotrimeric state of membrane-bound G_t_ was investigated. Here, high-salt extracts from dark-adapted ROS were used that contain an excess of free Gβ_1_γ_1_ (Fig. 1c, lane 5). As expected, light activation of ROS resulted in dissociation of heterotrimeric G_t_ and release of Gα_t_ into the high-salt extracts (Fig. 1c, lane 1). When ROS were washed with high-salt buffer containing a 2 molar excess of Nb5 with or without GTP, a similar release of Gα_t_ was observed. In contrast, high-salt extracts of ROS treated with Nb17 had no effect on either the heterotrimeric state of G_t_ or the release of Gα_t_ from ROS membranes (Fig. 1c, compare lanes marked with asterisks).

The effect of Nb5 on the G_t_ activation ability of Rh* was evaluated by monitoring the increase of intrinsic tryptophan fluorescence in Gα_t_ in the presence of Rh* at pH 7.0. Assay conditions were chosen such that the G_t_ activation rate was the same as that determined by GTPγS-induced complex dissociation^23^. A typical elevation of Gα_t_ intrinsic fluorescence was noted upon GTPγS-induced complex dissociation with Rh* (Fig. 1d). Interestingly, a 5 min pre-treatment of heterotrimeric G_t_ with a 2-fold molar excess of Nb5 resulted in a significant decrease in the G_t_ activation rate (Fig. 1d,e). In contrast, no significant effect was observed upon pre-treatment with Nb17 (Fig. 1d,e). This indicates that Nb5 alone can trap Gβ_1_γ_1_ after its spontaneous dissociation from Gα_t_, and thereby reduce the effective heterotrimeric population of G_t_ during G_t_ activation assays. Additionally, the observed changes in G_t_ activation kinetics with Nb5 are consistent with single turnover experiments of Rh* with different concentrations of heterotrimeric G_t_, that demonstrate a decrease in G_t_ activation rates with decreasing G_t_ concentrations (Fig. 1f). Overall, these results indicate that Nb5 has a high affinity towards the Gβ_1_γ_1_ dimer which shifts the dynamic equilibrium of heterotrimeric G_t_ towards dissociated Gα_t_ and Nb5-bound Gβ_1_γ_1_ complex in both solution and membranes.

### Nb5 competes with Gβγ regulatory proteins for Gβγ binding

**Fig. 2:**
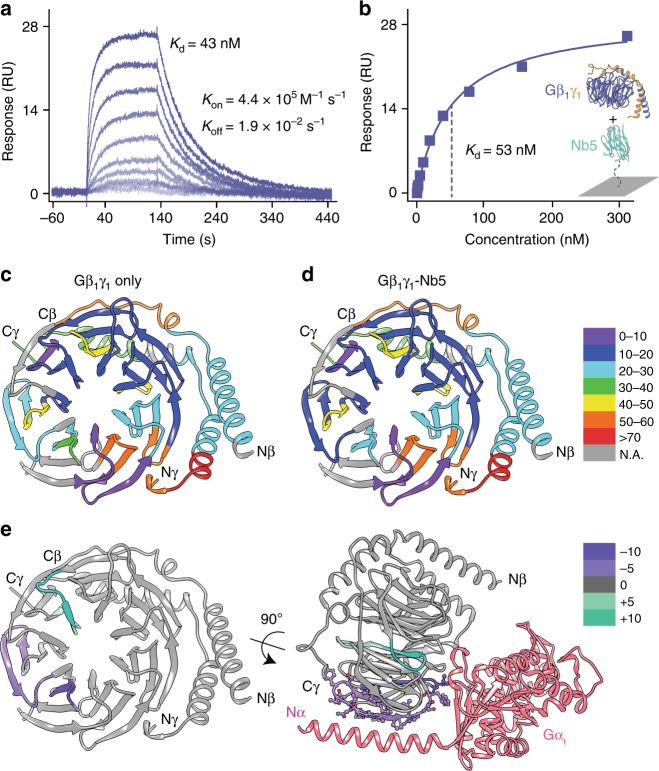
Kinetic profiling and hydrogen-deuterium exchange (HDX) of the Gβ_1_γ_1_-Nb5 complex. Binding of Gβ_1_γ_1_ to immobilized Nb5 in SPR equilibrium binding experiments. Association of the Gβ_1_γ_1_ dimer with Nb5 was investigated by single-cycle kinetics (**a**) and affinity based analysis (**b**). Resonance signals are indicated in response units (RU). The determined dissociation constants (*K*_d_) and kinetic parameters (*K*_on_ and *K*_off_) are shown as insets. HDX of the Gβ_1_γ_1_ dimer without (**c**) and with (**d**) Nb5 is shown with all the identified peptides colored by their percentage of deuterium exchange. **e** Differential HDX data mapped into the crystal structure of Gβ_1_γ_1_ (PDB ID: 5KDO) indicate peptides with increased (greencyan) and reduced (purple) deuterium incorporation upon Nb5 binding (left). Interestingly, Gβ_1_γ_1_ peptides that revealed changes in their solvent accessibility during HDX analyses displayed a partial interface with the Gα subunit (right, pink). The Gα subunit was omitted from the left panel for clarity

To examine the kinetics underlying the assembly of the Gβ_1_γ_1_-Nb5 complex, surface plasmon resonance (SPR) was employed with varied concentrations of native bovine Gβ_1_γ_1_. The binding kinetics analysis revealed a rapid on-rate constant (*K*_on_ = 4.4 × 10^5^ M^−1^ s^−1^) and a slow off-rate constant (*K*_off = _1.9 × 10^–2^ s^−1^) with a *K*_d_ of 43 nM (Fig. 2a). Analysis of the protein–protein interactions gave a similar *K*_d_ of 53 nM (Fig. 2b). The low-nanomolar affinity of Nb5 for Gβ_1_γ_1_ means a possible competition between Nb5 and other Gβ_1_γ_1_ regulatory proteins that bind Gβ_1_γ_1_ with similar affinities^24,25^. Differential HDX was used to investigate the binding dynamics between Gβ_1_γ_1_ and Nb5. Full HDX profiles of Gβ_1_γ_1_ alone were compared to those of the Gβ_1_γ_1_-Nb5 complex (Fig. 2c,d). Binding of Nb5 to the Gβ_1_γ_1_ dimer induced a statistically significant (*P* < 0.01) increase in protection from solvent exchange in the regions of residues 80–99 and 111–118 of the Gβ_1_ subunit versus the Gβ_1_γ_1_ dimer alone (Supplementary Table 1). In addition, regions with a statistically significant decrease in solvent protection were also observed (Fig. 2e, left). The differential HDX data indicate that the binding interface between Nb5 and the Gβ_1_γ_1_ dimer involves at a minimum, residues 80–99 and 111–118 of Gβ_1_ (Fig. 2e, left). Interestingly, the binding site of Nb5 on the Gβ_1_γ_1_ dimer implied by the HDX data closely resembles that of Gα_t_ with the Gβ_1_γ_1_ dimer (Fig. 2e, right). This suggests a possible binding interface overlap between Nb5 and Gα_t_ for the Gβ_1_γ_1_ dimer.

**Fig. 3:**
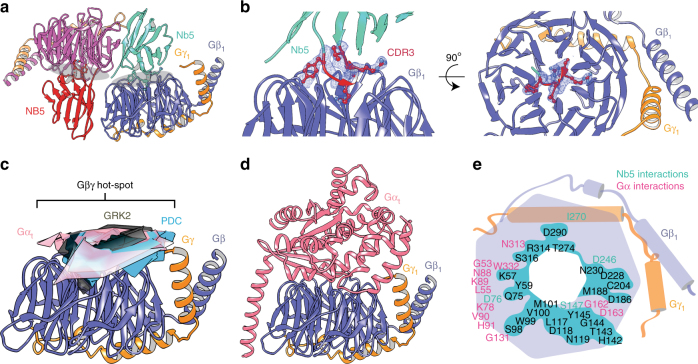
Crystal structure of Gβ_1_γ_1_ in complex with Nb5. **a** The asymmetric unit contains two molecules of both Gβ_1_γ_1_ (blue and pink) and Nb5 (greencyan and red) shown in cartoon representation. Key interfaces between Gβ_1_γ_1_ and Nb5 are denoted by grey ellipsoids. **b** Side and top views of the Gβ_1_γ_1_-Nb5 complex showing the CDR3 region (red) of Nb5 inserted into the Gβ_1_-propeller. The 2*|F*_*o*_*| - |F*_*c*_*|* density (blue) around the CDR3 region was calculated after the final refinement and is contoured at 1.5σ. **c** Gβγ hot-spot is shared by several Gβγ regulatory proteins, including Gα_t_ (pink), phosducin (PDC, cyan), and G Protein-Coupled Receptor Kinase 2 (GRK2, dark grey). **d** Overall structure of the heterotrimeric G protein (PDB ID: 5KDO). **e** Overlay of the interface between the Gβ_1_γ_1_-Nb5 complex and heterotrimeric G protein. Schematic representation shows the extent of overlapping (in greencyan background, black residues) between the non-canonical Gβ_1_γ_1_-Nb5 complex interface (greencyan residues) and the interface between Gβ_1_γ_1_ and Gα_t_ (pink residues)

To further investigate the binding between Gβ_1_γ_1_ and Nb5, the complex between Gβ_1_γ_1_ and Nb5 was crystallized. The crystal structure of the Gβ_1_γ_1_-Nb5 complex refined at 2.34 Å bore a strong resemblance to a heterotrimeric G protein structure. Two molecules of Gβ_1_γ_1_-Nb5 complex formed an asymmetric unit (Fig. 3a) wherein most of the crystallographic contacts were mediated by Nb5 (Supplementary Figure 2a). This agrees with a role of nanobodies as crystallization chaperones in the structural determination of challenging target proteins^26^. An intriguing feature of the Gβ_1_γ_1_-Nb5 complex is insertion of the CDR3 loop of Nb5 into the Gβ_1_-propeller, which occupies most of the binding interface (Fig. 3b). This finding is consistent with the nanomolar affinity observed in the SPR kinetics profiling of the Gβ_1_γ_1_-Nb5 complex (Fig. 2a, b). Interestingly, Nb5 occupies the same hot-spot region on the Gβ_1_γ_1_ dimer that is shared by other Gβγ regulatory proteins, including phosducin (PDC) and G protein-coupled receptor kinase 2 (GRK2) (Fig. 3a-c). Also, insertion of the Nb5 CDR3 loop into the Gβ_1_-propeller (interface area of 1030 Å^2^) is reminiscent of the interaction between the C-terminal loop of GRK2 and the Gβ_1_γ_2_ dimer (interface area of 1080 Å^2^; PDB accession: 1OMW^27^). Notably, Arg-101 in the Nb5 CDR3 loop serves as a key that locks the Gβ_1_-propeller. A similar interaction mechanism is mediated by Lys-663 of GRK2 in the Gβ_1_γ_2_-GRK2 complex (Supplementary Figure 2b,c). Complexation significance analysis^28^ that indicates the significance of a protein assembly formation showed a score of 0.27 for the Gβ_1_γ_1_-Nb5 complex versus 0.10 for the Gβ_1_γ_2_-GRK2 complex. The shape complementarity (Sc) index^29^ analysis of the binding interface formed by Nb5 and GRK2 with the Gβγ dimer displayed Sc scores of 0.80 and 0.57, respectively (1.0 is a perfect match). Although these results indicate that both Nb5 and GRK2 have a similar mode of binding with the Gβγ dimer, Nb5 is likely to have a structural advantage over GRK2. Similarly, Nb5 had a slight structural advantage over PDC for Gβ_1_γ_1_ binding (Sc index = 0.73). Interestingly, Arg-101 in the Nb5 CDR3 loop also forms an intricate hydrogen-bonding network with water molecules that extends throughout the Gβ_1_-propeller cavity (Supplementary Figure 2b). Consistent with the differential HDX analyses, the crystal structure of the Gβ_1_γ_1_-Nb5 complex revealed a significant overlap between the binding interfaces formed by Nb5 and Gα_t_ with Gβ_1_γ_1_ (Fig. 3b-e). Further, Sc analyses of the interface formed by Gα_t_ with Gβ_1_γ_1_ displayed a Sc index of 0.76 and an interface area of 1080 Å^2^ that are comparable to the Gβ_1_γ_1_-Nb5 complex. Overall, these results suggest a structural advantage of Nb5 over most Gβγ regulatory proteins except Gα, for Gβγ binding. Furthermore, a higher affinity of Gα (apparent *K*_d_ = 1–10 nM^30–32^) for the Gβγ dimer as compared to Nb5 (*K*_d_ = 43 nM) supports the inability of Nb5 to affect Gα signaling.

### Nb5 binds to various Gβ subtypes

**Fig. 4:**
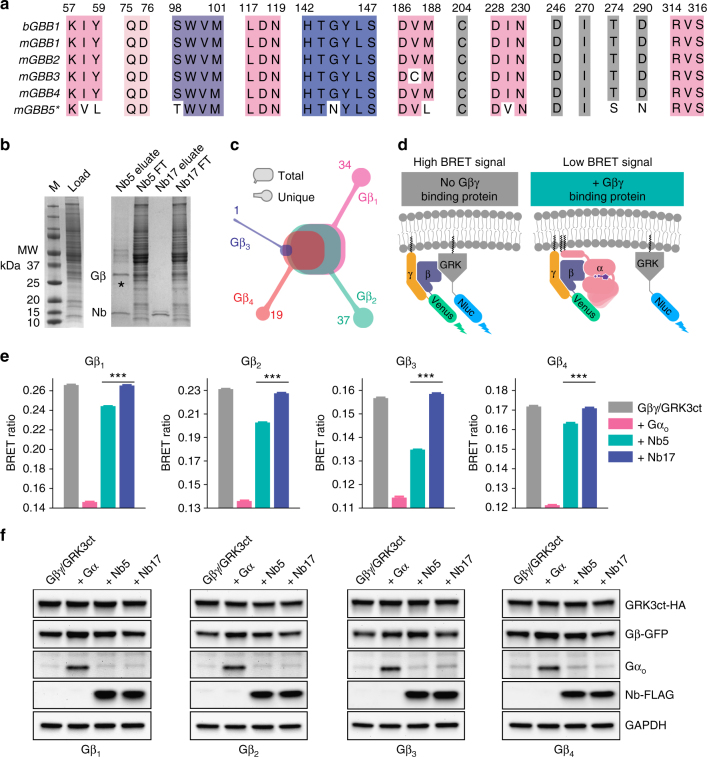
Gβ selectivity of Nb5. **a** Multiple sequence alignment of key amino acid residues of Gβ that interact with Nb5. *The amino acid numbering of Gβ_5_ has an off-set of −50. **b** Nb5-mediated Gβγ extraction from mouse brain. Solubilized and partially purified extracts from mouse brain (left) were subjected to either Nb5- (right, lanes 1 and 2) or Nb17- (right, lanes 3 and 4) mediated immobilized-metal affinity purification of Gβγ. **c** In-gel protein digestion of Gβ subtypes (band marked in **b** with asterisks) purified from mouse brain. Peptides were separated, analyzed and searched against a full mouse proteome to identify unique peptides from Gβ_1_, Gβ_2_, Gβ_3_, and Gβ_4_. **d** Schematic diagram of the effect of Gβγ-binding proteins on the BRET assay. Co-transfection of HEK293T/17 cells with Venus-Gβγ and masGRK3ct-Nluc-HA produced a high BRET signal through their direct interaction (left). Introduction of Gβγ-binding proteins (e.g., the Gα subunit) competed with masGRK3ct-Nluc-HA, lowering the BRET signal (right). **e** Effects of Nb5 on the interaction of Gβγ and the C-terminus of GRK3. The maximum BRET signal was determined by co-transfection of different Venus-Gβ subtypes + Gγ_2_ pairs and masGRK3ct-Nluc-HA (grey). A minimum BRET signal also was determined after co-transfection of Venus-Gβ_1_γ_2_ and masGRK3ct-Nluc-HA with an excess amount of Gα_oA_ (pink). Effects of Nb5 and Nb17 were examined by co-transfection of Venus-Gβγ and masGRK3ct-Nluc-HA with either Nb5 (greencyan) or Nb17 (purple). Experiments were performed with Gβ subtypes 1–4 + Gγ_2_ pairs. Each bar represents the mean of six replicates. Similar results were obtained in three independent experiments. Results are expressed as the mean ± SEM. One-way ANOVA with Tukey’s post hoc multiple comparison test relative to the Gβ_1_γ_2_/GRK3ct control, ***P ≤ 0.001, *n* = 6 replicates. **f** Western blot quantification of the expression levels of Gβ1–4, GRK3, Gα, Nb5, and Nb17 (Full blots are shown in Supplementary Figure 4a)

Assessment of the binding interface between the Gβ_1_γ_1_ dimer and Nb5 revealed critical amino acid residues that are highly conserved among Gβ subtypes 1–4 (Fig. 4a). To further investigate the putative binding of Nb5 with different Gβ subtypes, Nb5-mediated Gβ purification was carried out with C57BL/6J mouse brain. As expected, Nb5 bound and pulled out the different Gβ subtypes from mouse brain whereas Nb17 had no binding partner (Fig. 4b). In-gel protein digestion followed by mass-spectrophotometry (MS) analyses of the Gβ gel band (Fig. 4b, asterisks) identified unique peptides from Gβ_1_, Gβ_2_, Gβ_3_, and Gβ_4_ subtypes (Fig. 4c, Supplementary Table 3). However, unique peptides from the Gβ_5_ subtype were not observed due to its low sequence similarity with other Gβ subtypes. Notably, MS-identified peptides were searched against both the full mouse proteome and the primary sequences of Gβ subtypes to eliminate false-positives.

To further examine Gβ selectivity in a living cell, an optical assay was employed to monitor the interaction of Venus-tagged Gβγ and the nano Luciferase tagged C-terminal Gβ_1_γ_2_-interacting domain of G protein-coupled Receptor Kinase 3 (masGRK3ct-Nluc)^33^. Direct interaction of these sensor pairs increases the bioluminescence resonance energy transfer (BRET) ratio in transfected cells. As a result, co-transfection with a Gβγ-binding protein such as Gα, competes with the masGRK3ct-Nluc sensor and decreases the BRET ratio (Fig. 4d, pink). Indeed, exogenous Gα_o_ markedly diminished the BRET ratio (Fig. 4e, pink). Similarly, but to a lesser extent, Nb5 suppressed the BRET ratio in cells transfected with all Venus-Gβγ complexes tested (Fig. 4e, greencyan) suggesting an interaction between Nb5 and Gβ subtypes 1–4. In contrast, Nb17 had no effect on the BRET ratio suggesting no interaction with Gβ subtypes (Fig. 4e, purple). Additionally, western blot analyses of the transfected components were performed to verify similar expression levels of the exogenous proteins (Fig. 4f). Interestingly, the transfection of either Gα or Nb5, but not Nb17 slightly increased the expression levels of Venus-Gβ1–4 subunits, suggesting the formation of a proteolytically stable complex of Gα and Nb5 with Venus-Gβγ dimer. Overall, these experiments suggest that Nb5 binds to all combinations of Gβ subtypes 1–4 and Gγ and suppresses Gβγ signaling mediated through protein–protein interactions near the Gβγ hotspot. These results are suggestive of Nb5’s potential broad utility to influence Gβγ signaling in various cell types.

### Nb5 inhibits Gβγ-mediated GIRK signaling in striatum neurons

GIRK channels, found primarily in CNS neurons and atrial myocytes, respond to GPCR-mediated signaling through Gβγ binding events. Activation of GIRKs affects the flow of K^+^ ions across cell membranes that attenuate cellular electrical excitability. The capability of Nb5 to modulate Gβγ signaling was examined in medium spiny neurons (MSNs) from mouse striatum. GIRK channels (GIRK2; kir3.2) were virally overexpressed in the MSNs and the resulting outward currents were used as indicators of GPCR-mediated GIRK2 activation. Indirect pathway MSNs (iMSNs) expressed D2 dopamine receptors (D2Rs)^34^ whereas direct pathway MSNs (dMSNs) expressed M4 muscarinic acetylcholine receptors (M4Rs)^35^. Both D2Rs and M4Rs are G_i/o_-coupled receptors capable of gating GIRK channels through Gβγ signaling.

**Fig. 5:**
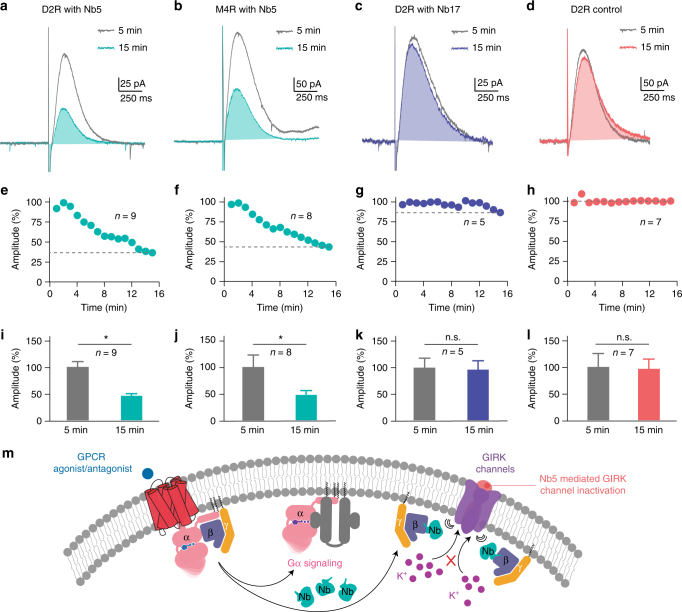
Inhibition of Gβγ-mediated GIRK signaling by Nb5. **a** Representative whole-cell recording of a GIRK2+ striatal medium spiny neuron (MSN) treated with 10 μM of intracellular Nb5. A single electrical stimulation evoked a D2R-IPSC that showed a significant amplitude reduction 15 min post-treatment with Nb5 (greencyan) as compared to the averaged trace observed at 5 min post-treatment with Nb5 (grey). **b** Representative trace of an M4-IPSC that displays an amplitude reduction 15 min post-treatment with Nb5 (greencyan) as compared to the averaged trace observed at 5 min post-treatment with Nb5 (grey). But no significant change was noted in D2R-IPSC either exposed to 10 μM intracellular Nb17 (**c**) or without nanobody treatment (**d**). Time course measurements showing a decrease in D2R-IPSC (**e**) and M4R-IPSC (**f**) amplitudes upon their exposure to 10 μM Nb5. Time course measurements of D2R-IPSC amplitudes recorded either with Nb17 (**g**) or without nanobody treatment (**h**). Bar graph quantification of D2R-IPSC (*n* = 9) (**i**) and M2R-IPSC (*n* = 8) (**j**) amplitudes recorded with Nb5. Bar graph quantification of D2R-IPSC amplitudes (*n* = 5) recorded either with 10 μM Nb17 (**k**) or without nanobody treatment (**l**), revealing no significant change in amplitudes over 15 min. Results are expressed as the mean ± SEM. *P < 0.05, Student’s *t*-test **m** Proposed model of GIRK inhibition by Nb5

A single electrical stimulation in the striatum evoked the release of dopamine from neuronal dopaminergic terminals, resulting in a D2R-mediated inhibitory post-synaptic current (D2R-IPSC) in postsynaptic GIRK2 positive iMSNs. Interestingly, inclusion of 10 μM Nb5 in the recording pipette attenuated D2R-IPSCs by about 60% (208.12 ± 47.08 pA in 1 min; 75.18 ± 9.40 pA after 15 min, *n* = 9, *P* < 0.05, Student’s *t*-test) (Fig. 5a, e, i). In contrast, including the irrelevant nanobody, Nb17 at the same concentration had no significant effect on the D2R-IPSCs amplitudes (210.05 ± 44.81 pA in 1 min; 186.66 ± 34.04 pA after 15 min, *n* = 5, *P* > 0.05, Student’s *t*-test) (Fig. 5c, g, k). Similarly, there was no effect on the D2R-IPSCs in postsynaptic GIRK2 positive iMSNs in the absence of nanobody treatment (271.50 ± 73.95 pA in 1 min; 257.20 ± 50.23 pA after 15 min, *n* = 7, *P* > 0.05, Student’s *t*-test) (Fig. 5d, h, l). To ensure that the effect of Nb5 was not D2R-specific, M4R-mediated inhibitory post-synaptic current (M4R-IPSC) measurements were also made on postsynaptic GIRK2 positive dMSNs. As expected, Nb5 attenuated M4-IPSCs evoked by electrical stimulation by about 60% (430.84 ± 81.09 pA in 1 min; 179.68 ± 22.70 pA after 15 min, *n* = 8, *P* < 0.05, Student’s *t*-test) (Fig. 5b, f, j). The effect of Nb5 in these GIRK inhibition assays was more demonstrable compared to its effects in the BRET assays. This likely is due to differences in the mode of delivery and thereby the effective concentration of Nb5 attained in cells in the two assay systems. While, Nb5 was co-transfected in human embryonic kidney cells 293 in BRET assays, GIRK recordings were performed by introducing an internal solution containing 10 μM of Nb5 directly to the neuronal exons. These results, together with the crystal structure of the Gβ_1_γ_1_-Nb5 complex demonstrate that Nb5 interferes with Gβγ dimer association to GIRK channels, thereby suppressing Gβγ-regulated GIRK signaling (Fig. 5m).

### Nb5 acts as a control-switch for GPCR-mediated Gβγ signaling

**Fig. 6:**
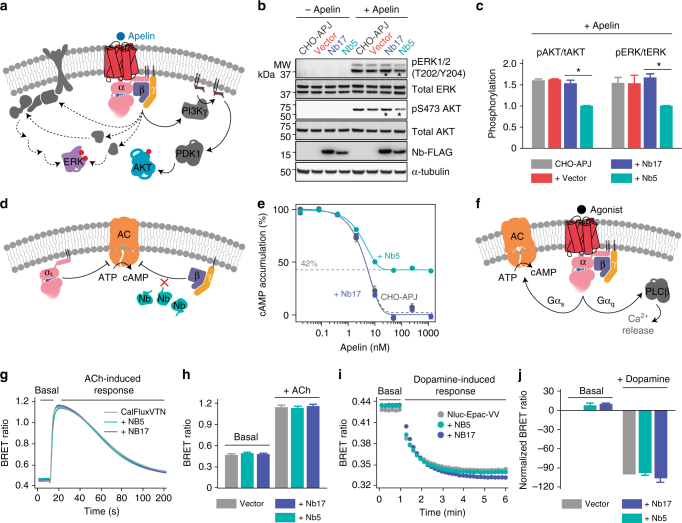
Nb5 as a control-switch for GPCR-mediated Gβγ signaling. **a** Schematic diagram of the GPCR-mediated Gβγ signaling network that controls various cellular functions. **b** Parental CHO-APJ cells, CHO-APJ cells transfected with pcDNA3.1 (+) empty vector, transfected CHO-APJ-Nb5 and CHO-APJ-Nb17 cells were treated with 1 μM apelin over 0–5 min to investigate the effect of Nb5 on the phosphorylation of ERK1/2 and AKT. Nb5 significantly decreased the phosphorylation of both ERK1/2 and AKT as compared to the Nb17 control (compare lanes marked with asterisks). Full blots are reported in Supplementary Figure 4b. **c** No significant effect of Nb17 transfection (purple) was observed on the phosphorylation of either ERK1/2 or AKT as compared to parental CHO-APJ cells (black) and CHO-APJ cells transfected with pcDNA3.1 (+) empty vector (red). Results are expressed as the mean ± SEM, *n* = 3 replicates, *P < 0.05, Student’s *t*-test. **d** Schematic diagram showing the effect of Gα_i_ and Gβγ signaling on cellular cAMP levels. **e** Dose-response curves of apelin-induced inhibition of cAMP accumulation in parental CHO-APJ cells (grey) and CHO-APJ cells expressing either Nb5 (greencyan) or Nb17 (purple). Apelin concentration is plotted on a semi-log scale. Results are expressed as the mean ± SEM, *n* = 4 replicates. **f** Schematic diagram of GPCR-mediated second-messenger regulation by Gα_q_ and Gα_s_. **g** The effect of nanobodies on Gα_q_ signaling events was determined in HEK293T/17 cells transfected with M3R and CalFluxVTN. Each trace represents the mean of the responses measured in six wells. **h** Basal BRET ratio and ACh-induced maximum amplitude shown as a bar graph. Similar results were obtained in three independent experiments. Results are expressed as the mean ± SEM, *n* = 6 replicates. Two-way ANOVA with Tukey’s post hoc multiple comparison test. **i** Effect of nanobodies on Gα_s_ signaling was evaluated in HEK293T/17 cells transfected with D1R and Nluc-Epac-VV. Each trace represents the mean of the responses measured in six wells. **j** Basal BRET ratio and dopamine-induced maximum amplitude shown as a bar graph. Each bar represents the mean of three independent experiments. Results are expressed as the mean ± SEM, *n* = 6 replicates. Two-way ANOVA with Tukey’s post hoc multiple comparison test

Next, the effect of Nb5 was investigated on cellular pathways that are often dysregulated such disease states as cancer. Here the PI3K-protein kinase B/AKT (PI3K-PKB/AKT)^17,18^ and MAPK-extracellular signal-regulated kinase (ERK)^20^ pathways were examined that are co-regulated by GPCR-mediated Gβγ signaling (Fig. 6a). The PI3K-PKB/AKT and MAPK/ERK pathways respond to a variety of cellular stimuli, and hence play a key role in diverse cellular processes, including cell survival, growth, proliferation, angiogenesis, metabolism, and migration. Because of the importance of these cellular pathways in cancer progression and metastasis, a Chinese hamster ovary cell line stably expressing human apelin receptor (APJ) was transfected with Nb5 cDNA and its effect upon the phosphorylation of AKT and ERK proteins downstream of APJ activation was assessed. Parental CHO-APJ cells were treated with 1 μM apelin over 0–30 min. Cell lysates then were collected and analyzed by immunoblotting to obtain optimum conditions to observe GPCR-mediated phosphorylation changes. Such changes were found most pronounced 5 min post-treatment with apelin (Supplementary Figure 3a). Interestingly, a significant decrease in the phosphorylation of both AKT and ERK1/2 proteins was observed in CHO-APJ-Nb5 cells 5 min post-treatment with apelin (Fig. 6b, c). In contrast, CHO-APJ cells, CHO-APJ-vector cells, and CHO-APJ-Nb17 cells showed no visible change in phosphorylation levels of either AKT or ERK proteins during the same time interval (Fig. 6b, compare lanes marked with asterisks). Additionally, no significant change was observed in the expression level of APJ with either Nb5 or Nb17 transfection (Supplementary Figure 3b). These results demonstrate a suppressive effect of Nb5 on APJ-mediated Gβγ signaling, further supporting its capability to modulate Gβγ-mediated pathways governed by multiple GPCRs.

APJ signaling is regulated by the G_i/o_ class of G proteins wherein both Gα_i_ and Gβγ act either together or separately to affect AC-mediated intracellular cAMP production (Fig. 6d)^36,37^. To determine the effect of Nb5 on intracellular cAMP levels, the forskolin-stimulated accumulation of cAMP was measured in response to apelin treatment. Treatment of CHO-APJ cells with apelin inhibited the forskolin-stimulated accumulation of cAMP in a dose-dependent manner (Fig. 6e). CHO-APJ cells co-expressing Nb5 (greencyan) showed a reduced inhibition of cAMP accumulation at a higher dosage of apelin when compared to either parental CHO-APJ cells (grey) or CHO-APJ cells expressing the negative control Nb17 (purple). This result demonstrates that Nb5 modulates the apelin-induced inhibition of cAMP accumulation in CHO-APJ cells. In addition, Nb5 presumably had no effect on the Gα_i/o_-GTP-mediated cAMP signaling after the loss of the Gβγ-mediated cAMP component in cells transfected with Nb5 (Fig. 6e, greencyan, Supplementary Table 4). These results also are consistent with the higher affinity of Gα compared to Nb5 for the Gβγ dimer. Overall, the binding of Nb5 to Gβγ negatively regulates G_i/o_ signaling, at least partially by inhibiting Gβγ-associated cAMP signaling.

To further confirm the specificity of Nb5 towards Gβγ-induced signaling, we tested its effects on two well-established agonist-induced Gα signaling pathways (Fig. 6f)^33^. Here, Gα_q_ signaling was monitored in HEK293T/17 cells transfected with the M3 muscarinic acetylcholine receptor (M3R) and using a Ca^2+^ sensor (CalFluxVTN). Gα_s_ signaling was monitored in HEK293T/17 cells transfected with the dopamine D1 receptor (D1R) and using a cAMP sensor (Nluc-Epac-VV). The transfected cells were then stimulated with acetylcholine or dopamine to activate M3R or D1R, respectively. Real-time monitoring of the resultant responses clearly demonstrated that Nb5 had no effect on the elevation of intracellular Ca^2+^ induced by the activation of M3R-Gα_q_ (Fig. 6g, h) or on D1R-Gα_s_-induced cAMP production (Fig. 6i, j) in living cells.

## Discussion

The significance of Gβγ signaling in various cellular functions has received appropriate recognition only recently in comparison to Gα signaling. The Gβγ dimer functions as a negative regulator when bound to the Gα subunit. Additionally, the Gβγ dimer regulates many downstream events depending on its interaction with different effector molecules. Many of these downstream events are dysregulated in neurological disorders and cancer, making the Gβγ dimer a critical drug target. However, the ability of Gβγ subunits to play essential roles in various cellular functions, including the formation of heterotrimeric G proteins, implies the potential for numerous side effects when Gβγ is the pharmacological target. In this study, it was found that Nb5, a nanobody against Gβ_1_, can cause selective inhibition of Gβγ-mediated signaling while leaving GTP-bound Gα_q_ and Gα_s_-mediated signaling intact. G_t_ activation analyses showed that Nb5 causes a GPCR-independent shift in the dynamic equilibrium of heterotrimeric G_t_ into its dissociated subunits in both membranes and in solution. Both in vitro SPR kinetics and G_t_ activation assays suggest a tight interaction between Nb5 and the Gβγ dimer. Gβγ binding proteins, including Gα, PDC, GRKs, and PI3K are key regulators of GPCR signaling known to interact with the Gβγ dimer with nanomolar affinities. A similar binding affinity of Nb5 with Gβ_1_γ_1_ ensures competition between Nb5 and these Gβγ regulatory proteins in modulating Gβγ-mediated signaling events. Indeed, combined differential HDX and x-ray crystallography studies demonstrate a possible competition between Nb5 and Gβγ regulatory proteins due to their overlapping binding interfaces. In fact, Sc index analysis that precisely measures correlations of directions suggests a structural advantage of Nb5 over most Gβγ regulatory proteins except Gα for Gβγ binding. Furthermore, the binding interface between Nb5 and Gβ_1_γ_1_ has the highest Sc index among several tested protein-Nb interactions^38,39^. Nb5-mediated Gβ purification from mouse brain and cell-based BRET assays demonstrate the specificity of Nb5 towards Gβ subtypes 1–4. No evidence for binding between Nb5 and Gβ_5_ was observed, likely because the sequence of Gβ_5_ differs from that of other Gβ subtypes with only 53% sequence identity to its closest Gβ subtype versus 80–90% sequence identity between other Gβ subtypes. Overall, these results identify Nb5 as a potential negative regulator of Gβγ signaling in various cell types.

The M119 class of small-molecule inhibitors, which includes M119, gallein and M119K, are potent Gβγ antagonists. They remain the most extensively validated inhibitors of Gβγ signaling. Studies to date indicate that these molecules bind to a Gβγ hot spot on the top surface of the β subunit with apparent affinities in the high nM to low μM range^40^. The M119 class of inhibitors displays a limited number of chemical moieties that interact with the Gβγ dimer. Therefore, structure activity relationship studies to achieve Gβ selectivity would have limited practicality. Nb5, on the other hand, binds Gβγ with a low nM affinity and has a larger area of interaction with the Gβγ dimer (>1030 Å^2^). These features indicate that Nb5 might serve as a better template for structure activity relationship studies to achieve Gβ selectivity. Unlike nanobodies, the M119 class of inhibitors can be delivered orally and intraperitoneally to modulate Gβγ-dependent pathways, which admittedly takes them one step closer to clinical applications. In addition, the M119 class of inhibitors has the advantage that they specifically target the formation of Gβγ-GRK2 complexes^40^. Challenges remain for the development of nanobody-based clinical therapies due to the dependence of this approach on efficient gene transduction methods. Moreover, the ability of Nb5 to affect general Gβγ signaling raises concerns for possible off-target effects when pursued therapeutically^41^. Nevertheless, there may be applications for Nb5-based treatments where the beneficial effects outweigh the side effects. The C-terminal domain of GRK2 (βARK-ct), PDC and several affinity matured peptides, including the SIRK peptide family, comprise an alternative class of potent Gβγ inhibitors that are mechanistically similar to Nb5^42–44^. While βARK-ct and PDC have a large area of interaction with the Gβγ dimer (>1000 Å^2^), no atomic level information is available for SIRK or other peptides except that they bind to the same Gβγ hot spot with high nM affinities^44,45^ and selectively inhibit the binding of Gβγ effector molecules. Several cell-permeating versions of SIRK peptides have been shown to effectively modulate ERK1/2 and MAPK signaling in arterial smooth muscle cells^43^. Treatment strategies involving Nb5, βARK-ct, PDC, and members of the SIRK peptide family would all require gene transduction methods, however this may be worthy of significant effort given their potential to treat various Gβγ-related disorders.

Many neurological and neuromuscular disorders including Parkinson disease, Alzheimer disease, bipolar disorder, multiple sclerosis, and other age-related disorders are aggravated by dysregulated ion channel function. Ion channels like GIRK play a crucial role in maintaining ion homeostasis in neurons, determining their membrane potential and neurotransmitter secretion. The lack of endogenous GIRK channels in MSNs and their effective coupling to various GPCR signaling pathways makes them a useful tool for rapid sensing and monitoring of Gβγ-regulated GIRK signaling^35^. Whole-cell patch clamping of GIRK2 overexpressed MSNs was performed to investigate the role of Nb5 as a negative regulator of Gβγ-mediated signaling. Treatment with Nb5 decreased both D2R- and M4R-IPSCs amplitudes, and thus inhibited Gβγ-regulated GIRK channel opening. This serves as one of the first examples wherein a nanobody modulates a GPCR-mediated Gβγ-signaling event in any cell type. A body of evidence demonstrates that Gβγ subunits play significant roles in regulating downstream G protein signaling including the MAP kinase pathway. Previous studies have shown that Gβγ antagonist βARK1-(495–689) inhibits Gα_i_-mediated ERK activation in a Gβγ dependent manner^46,47^. Extensive studies on small molecule-based and peptide-based Gβγ inhibitors show that Gi-coupled GPCR-mediated ERK signaling is altered by M119 class and SIRK Gβγ inhibitors^40,43^. Our data also suggest that Nb5 reduces downstream phosphorylation events in Gα_i_-linked AKT and ERK kinase pathways. Notably, Nb5 does not completely ablate either GIRK activation or downstream AKT and ERK phosphorylation, suggesting its capability to suppress dysregulated pathways with critical roles in cancer progression and metastasis.

The Gβγ dimer classically was thought to inhibit AC activity by binding and inhibiting stimulatory Gα subunits. However, recent studies have shown that Gβγ can stimulate several AC isoforms through cross-talk between Gα-mediated and Gβγ-mediated cAMP signaling^19,48,49^. Co-expression of Nb5 in CHO-APJ cells showed a 58% reduction in apelin-mediated inhibition of cAMP accumulation when compared to either parental cells or CHO-APJ co-expressing Nb17. This result demonstrates that Nb5 modulates the forskolin-stimulated accumulation of cAMP in CHO-APJ cells by inhibiting the Gβγ-mediated cAMP signaling component. Additionally, Nb5 showed no significant effect on either the M3R-Gα_q_-induced intracellular Ca^2+^ elevation or D1R-Gα_s_-induced cAMP production. These results show that Nb5 does not alter the GTP-bound Gα_q_ and Gα_s_-mediated signaling component in living cells. Overall, Nb5 serves as a dynamic scavenger of the Gβγ dimer and thereby causes partial inhibition of Gβγ-mediated signaling. The remaining Nb5-free Gβγ supports the canonical Gα-GTP-mediated signaling that includes the GPCR-mediated GDP-GTP exchange from Gα, and signaling termination by GTP hydrolysis and re-association of Gα-GDP with Gβγ. However at higher concentrations, Nb5 might affect the Gα-GTP signaling component due to increased sequestration of Gβγ dimers (Fig. 1d,e).

In summary, the ability of nanobodies to act as crystallization chaperones by either stabilizing intrinsically flexible regions or shielding aggregating surfaces in a protein is well established^50^, but their ability to serve functional roles in cellular signaling has not been well studied. This work highlights the functional importance of these genetically-encodable antibody fragments in controlling various aspects of GPCR-signaling pathways. Interestingly, Nb5 acts as a specific inhibitor of various Gβγ-mediated signaling events that regulate several ‘undruggable’ targets such as GIRK channels, ERK, and AKT kinases. The ability of Nb5 to suppress but not completely ablate Gβγ signaling makes it a beneficial tool for future nanobody-based therapeutics to modulate cellular signaling. This work on Nb5 provides a proof of principle and serves as one of the first examples wherein a nanobody modulates a GPCR-mediated Gβγ-signaling event in any cell type. Future experiments will focus on increasing the Gβ selectivity of Nb5 by using both structure guided mutational studies and development of Gβ selective phage display libraries. The ease of production and genetic manipulation of nanobodies are advantageous characteristics for achieving the goal of engineering variants with specificity toward different Gβ subtypes and even Gβγ combinations. This opens up the possibility of designing targeted treatments for various excitatory neurological conditions and inhibitors for blocking cancer progression. However, the development of nanobody-based clinical therapies relies heavily on the advancement of specific and efficient gene transduction methods such as CRISPR/Cas9 and viral-gene transduction. Additional strategies for intracellular nanobody delivery include coupling the Nbs to cell-penetrating peptides such as penetratin^51^. Elucidating nanobody translocation across the CNS could also provide a tremendous advantage in treating neurological disorders. Such strategies might include internalization via either clathrin-coated vesicles^52^ or Trojan horse technology using transferrin and insulin receptors^53^. The comprehensive biochemical and structural analyses of Nb5 reported in this study provide a basis for further research studies to turn nanobodies into a viable class of therapeutics. We believe that nanobody-based therapeutics will find a niche in cases where ‘undruggable’ protein targets are involved in multiple signaling pathways and where conventional therapeutic approaches induce unacceptable side effects.

## Methods

### Reagents

Antibodies recognizing phosphorylated-ERKs 1/2 (αpT202/pY204-ERK1/2, catalog no. 9106), total ERKs 1/2 (αERK1/2, catalog no. 4695), phosphorylated-Akt (αpS473-Akt, catalog no. 4058), total Akt (αAkt, catalog no. 9272), and anti-α-tubulin (catalog no. 2144) were from Cell Signaling Technology (Danvers, MA). Anti-APJ antibody (3C3–7, catalog no. MABN1846) was from Millipore Sigma (St. Louis, MO). Polyclonal αFlag antibody (catalog no. A190-101A) was from Bethyl Laboratories, Inc. (Montgomery, TX). Anti-Flag antibody (catalog no. F7425), αHA antibody (catalog no. 11867423001), αGAPDH antibody (catalog no. MAB374) and αGFP antibody (catalog no. 11814460001) were from Millipore Sigma (St. Louis, MO) and used for Western blotting analysis in Fig. 4f. Anti-Gαo antibody (catalog no. sc-387) was from Santa Cruz (Dallas, TX). IRDye-conjugated anti-mouse IgGs (catalogs P/N 925-32210 and P/N 925-68070), and anti-rabbit IgGs (P/N 925-32211 and P/N 925-68071) were from LI-COR (Lincoln, NE). HRP-anti-goat IgG was from ThermoFisher Scientific, Inc (Waltham, MA). Apelin-13 (catalog no. 057-18) was from Phoenix Pharmaceuticals, Inc (Burlingame, CA). IBMX (catalog no. 2845) and Forskolin (catalog no. 1099) were from R&D systems, Inc (Minneapolis, MN). CHO-K and HEK293T/17 cells (catalog nos. CRL-9618 and CRL11268, respectively) were from the American Type Culture Collection (Manassas, VA) and have no mycoplasma contamination. All antibodies were used at 1:1000 dilution.

A pCMV5 plasmid encoding Gα_oA_ was a gift from Dr. Hiroshi Itoh (Nara Institute of Science and Technology, Japan). Plasmids encoding Venus 156-239-Gβ_1_, and Venus 1-155-Gγ2 were gifts from Dr. Nevin A. Lambert (Augusta University)^54^. The masGRK3ct-Nluc-HA constructs were constructed by introducing HA tag at the C-terminus of masGRK3ct-Nluc reported previously^33^. Amino acids 156–239 of Venus were fused to a GGSGGG linker at the N-terminus of Gβ_2_ (GenBank: AF501883), Gβ_3_ (GenBank: M31328), or Gβ_4_ (GenBank: AF300648) to construct Venus 156–239-Gβ subunits. Nb5-Flag and Nb17-Flag constructs were based on the primary sequences of Nb5 and Nb17 wherein the 6 × His-tag was replaced by the FLAG-tag (GATTACAAGGATGACGACGATAAGTAG). Human M3 muscarinic acetylcholine receptor and human dopamine D1 receptor in pcDNA3.1(+) were purchased from cDNA Resource Center (Bloomsberg, PA). Nluc-Epac-VV and CalFluxVTN in pcDNA3.1(+) were reported previously^33^. Adeno-associated virus (AAV) AAV9.hSyn.tdTomato.T2A.mGIRK2-1-A22A.WPRE.bGH was obtained from the University of Pennsylvania Viral Core^35^.

### Generation and phage selection of nanobodies

Nanobodies against the Gβ_1_γ_1_ dimer were prepared by a previously published protocol^10^. Briefly, a llama (Lama glama) was immunized weekly for six weeks with 1 mg of purified bovine Gβ_1_γ_1_ dimer. Peripheral blood lymphocytes from anti-coagulated blood were then used to prepare cDNA clones that served as templates for amplification of open reading frames encoding the variable domains of the heavy-chain only antibodies. PCR fragments were then ligated into the pMESy4 phage display vector and transformed in *E. coli* TG1 cells (Lucigen, Middleton, WI) to create a nanobody library of 4 × 10^9^ transformants. After super infection with M13 helper phage^10^, the display library was added to antigen-coated wells of MaxiSorp ELISA plates and selections were performed in buffer containing 10 mM HEPES, pH 7.5, 100 mM NaCl, 2 mM MgCl_2_, 1 mM EDTA, and 1 mM DTT. After washing, phage were eluted by incubating the antigen-coated wells with 100 µl of trypsin (250 µg ml^−1^) for 30 min. Freshly grown TG1 cells were then infected with the eluted phage and grown overnight at 37 °C. A total of 184 colonies were randomly picked and analyzed with ELISA. Testing for specific binding to the Gβ_1_γ_1_ dimer resulted in 14 families wherein all nanobodies were produced as soluble His-tagged proteins in the *E. coli* periplasmic region^10^.

### Nanobody expression and purification

Nanobodies (Nb5 and Nb17) were expressed and purified as described before^10^. Briefly, nanobodies bearing a C-terminal His-tag were transformed in *E. coli* WK6 (Su^−^) cells^55^. Small scale cultures of 5 ml were grown in LB media containing 2% glucose, 1 mM MgCl_2_ and 50 μg ml^−1^ ampicillin. Large scale cultures of 2 L were grown to OD_600_ = 0.9–1.0 at 37 °C in TB media containing 0.1% glucose, 1 mM MgCl_2_, and 50 μg ml^−1^ ampicillin. Cultures were induced with 1 mM IPTG and grown overnight at 28 °C. Cells were harvested by centrifugation at 11,000 *g* for 20 min at 4 °C. The periplasmic fraction was extracted by re-suspending cells in ice-cold TES buffer containing 0.2 M Tris, pH 8.0, 0.5 mM EDTA, and 0.5 M sucrose. Following a 1 h incubation at 4 °C, cells were supplemented with 4 × diluted TES buffer to achieve a final buffer composition of 0.1 M Tris, pH 8.0, 0.25 mM EDTA, and 0.25 M sucrose. After 1 h incubation at 4 °C, cells were removed by centrifugation at 11,000 *g* for 30 min at 4 °C. The periplasmic extract was filtered through a 0.22 μm vacuum driven filter and subjected to immobilized-Ni^2+^ affinity chromatography followed by size exclusion chromatography (SEC) on a Superdex 200 10/300 GL column equilibrated with 20 mM bis-Tris propane, pH 7.0 and 100 mM NaCl. Peak protein fractions were pooled and concentrated to 10 mg ml^−1^.

### Rh purification

All experimental procedures were carried out in a darkroom under dim red light (>670 nm). Bovine rod outer segments (ROS) were prepared as described elsewhere^34^. ROS were washed with isotonic and hypotonic buffer to remove both soluble and membrane-associated ROS proteins^56,57^. Rh was purified by a protocol described previously^58^. Briefly, native Rh membranes were solubilized by a zinc/alkyl-glucoside extraction method and centrifuged at 100,000 *g* for 40 min to extract Rh. Clear supernatants were loaded on a 1D4-coupled CNBr-activated Sepharose 4B column and washed with buffer containing 10 mM MES, pH 6.4, 100 mM NaCl, and 0.02% *n*-dodecyl β-D-maltoside (DDM) to either dispose of excess retinal or achieve further purification. Finally, purified Rh was eluted with 0.5 mg ml^−1^ of TETSQVAPA nanopeptide (from the Rh C-terminal sequence).

### Purification of heterotrimeric G_t_

Bovine rod outer segments (ROS) were prepared as described elsewhere^34^ in a darkroom under dim red light (>670 nm). ROS were resuspended in isotonic buffer containing 20 mM HEPES, pH 7.5, 100 mM NaCl, 1 mM DTT and 5 mM MgCl_2_. Resuspended ROS were then centrifuged at 31,000 *g* at 4 °C for 25 min to remove soluble and some membrane-associated proteins. The pellet was then gently homogenized twice in hypotonic buffer containing 5 mM HEPES, pH 7.5, 1 mM EDTA, and 1 mM DTT by manually passing the solution through a glass-to-glass homogenizer. The homogenized suspension was centrifuged at 40,000 *g* for 30 min at 4 °C. Supernatants from the two hypotonic washes were pooled and centrifuged multiple times at 40,000 *g* for 30 min at 4 °C to completely remove any residual ROS pellet. The clear supernatant was dialyzed against the equilibrating buffer containing 10 mM HEPES, pH 7.5, 2 mM MgCl_2_, and 1 mM DTT for 3 h at 4 °C. For purification of heterotrimeric G_t_, the hypotonic solution was loaded onto a C10/10 column (GE Healthcare) with 6 mL of pre-equilibrated propyl-agarose resin. Next, the column was washed with 30 resin volumes of the equilibration buffer followed by 2 resin volumes of the same buffer containing 50 mM NaCl. Bound proteins were eluted with 50 mL of equilibration buffer containing 0.5 M NaCl. The eluate containing heterotrimeric G_t_ was concentrated and loaded onto a Superdex 200 10/300 GL column equilibrated with equilibration buffer containing 0.1 M NaCl. Fractions containing heterotrimeric G_t_ were combined, concentrated to about 2 mg ml^−1^ and used for further analyses.

### Nanobody-mediated shift in heterotrimeric G_t_ equilibrium

ROS were resuspended in isotonic buffer containing 20 mM HEPES, pH 7.5, 100 mM NaCl, 1 mM DTT, and 5 mM MgCl_2_. For G_t_ dissociation experiments in ROS membranes, the resuspended ROS (2 mg ml^−1^ Rh) were either illuminated with a 150-W fiber light in the presence of 250 μM GTP, co-treated with 250 μM GTP and 3 μM Nb5, or treated with either 3 μM Nb5 or 3 μM Nb17 for 30 min at 4 °C. The resuspensions were then centrifuged at 100,000 *g* at 4 °C for 20 min and the supernatants were analyzed by SDS-PAGE. In solution G_t_ dissociation experiments were conducted on supernatants obtained after ROS hypotonic washes. Hypotonic extracts were combined and centrifuged multiple times at 40,000 *g* for 30 min at 4 °C to ensure complete removal of the residual ROS pellet. Next, the hypotonic extracts (1 μM G_t_) were either supplemented with 1 μM Rh and illuminated with a 150-W fiber light in the presence of 250 μM GTP and 2 μM Nb5, co-treated with 250 μM GTP and 2 μM Nb5, or treated with either 2 μM Nb5 or 2 μM Nb17 for 30 min at 4 °C. Then these treated hypotonic extracts were subjected to immobilized-Ni^2+^ affinity chromatography. A small volume of Ni^2+^-NTA resin (250 μl) pre-equilibrated with 10 mM HEPES, pH 7.5, 0.1 M NaCl, 2 mM MgCl_2_, and 1 mM DTT was added to the treated hypotonic extracts. After 1 h of incubation, the resin was washed with 50 resin volumes of equilibration buffer and bound proteins were eluted in equilibration buffer containing 300 mM imidazole, pH 7.5. Eluted fractions were then analyzed by SDS-PAGE.

### G_t_ activation assay

The intrinsic fluorescence increase from Gα_t_ was measured with a L55 luminescence spectrophotometer (PerkinElmer Life Sciences) operating at excitation and emission wavelengths of 300 and 335 nm, respectively^23,59^. The ratio of G_t_ to Rh was 20:1, with G_t_ at a concentration of 1000 nM and Rh at 50 nM. G_t_ was pre-incubated with a two molar excess of either Nb5 or Nb17 to determine their effects on Rh*-mediated G_t_ activation rates. This was followed by the addition of 300 µM GTPγS (Sigma-Aldrich) to determine the GTPγS-induced complex dissociation and fluorescence changes^58^. Samples were bleached for 1 min with a Fiber-Light delivered through a 480–520 nm long pass wavelength filter prior to the fluorescence measurements. G_t_ activation rates were determined for the first 100 s in a G_t_ activation assay^58^.

### Gβ_1_γ_1_ alone and Gβ_1_γ_1_-Nb5 complex purification

Bovine ROS were washed with isotonic buffer containing 20 mM HEPES, pH 7.5, 100 mM NaCl, 1 mM DTT, and 5 mM MgCl_2_. For Gβ_1_γ_1_ purification, ROS washed with isotonic buffer were illuminated with a 150-W fiber light (NCL-150, Volpi, USA) for 15 min and then washed twice with hypotonic buffer containing 5 mM HEPES, pH 7.5, 1 mM EDTA, and 1 mM DTT supplemented with 250 μM GTP. The hypotonic wash extracts were pooled and applied to a Blue Sepharose CL-GB column pre-equilibrated with 10 mM HEPES, pH 7.7, 6 mM MgCl_2_, 1 mM EDTA, and 1 mM DTT. Gβ_1_γ_1_ obtained from the unbound flow-through was further purified by SEC on a Superdex 200 10/300 GL column (GE Life sciences) equilibrated with 50 mM Tris-HCl, pH 7.5, 100 mM NaCl, 2 mM MgCl_2,_ and 3 mM DTT. For purification of the Gβ_1_γ_1_-Nb5 complex, ROS washed with isotonic buffer were washed twice with hypotonic buffer and the washes were combined for further purification. Hypotonic wash extracts were then incubated with a 1:2 molar ratio of Nb5 for 1 h at 4 °C. This was followed by the addition of Ni^2+^-NTA resin pre-equilibrated with 50 mM Tris-HCl, pH 7.5, 100 mM NaCl, 5 mM imidazole, 2 mM MgCl_2_, and 1 mM DTT. Following 1 h of incubation, the resin was washed with 50 resin volumes of equilibration buffer and the Gβ_1_γ_1_-Nb5 complex was eluted with equilibration buffer containing 300 mM imidazole. The Ni^2+^-NTA purified Gβ_1_γ_1_-Nb5 complex was then loaded on a Superdex 200 10/300 GL column equilibrated with 50 mM Tris-HCl, pH 7.5, 100 mM NaCl, 2 mM MgCl_2_, and 3 mM DTT.

### Gβ_1_γ_1_-Nb5 complex crystallization

SEC fractions were concentrated to 4.9 mg ml^−1^ and then used for crystallization. The concentrated protein was supplemented with 2 mM DTT. Crystallization screens by the sparse matrix crystallization method were carried out by both the hanging-drop and sitting drop vapor diffusion methods. Each hanging drop was prepared on a siliconized coverslip by mixing equal volumes of Gβ_1_γ_1_-Nb5 complex and reservoir solution. The reservoir solution contained 25% (w/v) PEG 3350 in 0.1 M Bis-Tris-HCl, pH 5.5–5.7, and 0.2–0.3 M MgCl_2_. Crystals appeared in 2 days at 4 °C and reached 50–80 μm in their longest dimension within 5 days. Crystals were harvested directly from the mother liquor into dual-thickness microloops (MiTeGen, LLC) and plunge-frozen in liquid nitrogen.

### Diffraction data collection and structural refinement

X-ray data collection of the Gβ_1_γ_1_-Nb5 complex was performed at −173 °C. Diffraction data were collected at the NE-CAT-24-ID-E beamline. Data were integrated with XDS and scaled using XSCALE^60^. Initial phases for the Gβ_1_γ_1_-Nb5 complex were obtained by molecular replacement using the Gβ_1_γ_1_-phosducin complex and TssK nanobody nb18 structures as search models (PDB accession: 1A0R^21^, 5M2W^61^) with the CCP4 program PHASER^62,63^. Initial models were improved by multiple rounds of REFMAC ver. 5.8^62^ refinement against the Gβ_1_γ_1_-Nb5 complex dataset and manual model adjustments with Coot 0.8.8^64^. The final models had agreement factors R_*free*_ and R_*cryst*_ of 24.7% and 20%, respectively. The stereochemical quality of the Gβ_1_γ_1_-Nb5 complex model was assessed with the Molprobity^65^ and wwPDB validation servers^66^. Details of the diffraction data collection and structural refinement statistics are provided in Supplementary Table 2. Coordinates and structure factor amplitudes were deposited in the PDB (PDB accession: 6B20). The dimer interface shape complementarity was calculated by Sc^29^ and PISA^28^.

### Differential HDX

Amide H/D exchange was performed as described previously^67^. Briefly, 10 μg of purified Gβ_1_γ_1_ or Gβ_1_γ_1_-Nb5 complex were diluted in 70 μL of ice cold D_2_O to obtain a final concentration of 80% D_2_O. The solution was incubated on ice for 10 min to achieve steady state deuterium exchange conditions. This was followed by quenching the reaction with ice cold buffer containing D_2_O in formic acid (Sigma-Aldrich) to obtain a final pH of 2.5. Non-deuterated samples were quenched with buffer containing H_2_O in formic acid, pH 2.5. Samples were digested with freshly prepared 5–20 μg pepsin (Worthington, Lakewood, NJ) solution prepared in H_2_O. All samples were digested over a time range of 1–5 min on ice. Next, samples (100 μL) were loaded onto a C8 trap (2.1 mm, Thermo Scientific) and a C18 (20 mm × 2.0 mm, Phenomenex) column using a temperature-controlled Accela 600 autosampler and pump (Thermo Scientific) with a temperature set to 4 °C. Separation was achieved with an Agilent 1100 HPLC system (Agilent Technologies, Santa Clara, CA) at a flow rate of 0.1 ml min^−1^. Peptides were eluted with a gradient from 98% of buffer A containing 0.1% (v/v) formic acid in H_2_O and 2% of buffer B containing 0.1% (v/v) formic acid in acetonitrile to 2% of buffer A and 98% of buffer B. The eluent was injected into a Thermo Finnigan LXQ (Thermo Scientific, Waltham, MA) MS equipped with an electrospray ionization source operated in the positive ion mode with other parameters adjusted as follows: activation type was set to collision induced dissociation, normalized collision energy to 35 kV, capillary temperature to 370 °C, source voltage to 5 kV, capillary voltage to 43 V, tube lens to 105 V, and then MS spectra were collected over a 200–2000 mass range. To avoid contamination from previous runs, each production run was followed by a 10 μl mock injection of buffer A, followed by residual peptide elution with the gradient profile described above. Each run was also followed by a 20 min equilibration run with 98% buffer A and 2% buffer B.

### Differential HDX data analysis

mzXML files were generated from raw data with the MassMatrix file conversion tool. Peptides were identified by searching against the primary sequence of bovine G protein beta 1 (*GBB1*; Uniprot ID: P62871) and G protein gamma 1 (GNGT1**;** Uniprot ID: P02698) using MassMatrix v3.10. Search parameters for peptide identification were as follows: precursor ion tolerance, 3.0 Da; variable modifications, farnesylation and methylation of the N-terminus; minimal peptide length, 6 amino acids; minimal pp score, 5; pptag score, 1.3; maximal number of combinations of different modification sites for a peptide match with modifications, 1; and maximal number of candidate peptide matches for each spectrum output in the result, 1. Raw data in the form of the relative signal intensity (percent) as a function of *m/z* were extracted with Xcalibur version 2.1.0. Qual Browser was used for recently described semiautomated peak detection, and a deconvolution procedure was performed with HXExpress^68^.

### SPR analyses

SPR data acquisition was carried out using the Biacore T100 SPR instrument (Biacore, GE Healthcare) at 25 °C. Purified Nb5 (50 ng ml^−1^) was injected at a flow rate of 10 μl min^−1^ in 10 mM sodium acetate, pH 5.5 to achieve capture level between 400–500 resonance units (RU) on a CM5 sensor chip according to directions in the manufacturer’s amine coupling kit. After Nb5 immobilization, the surface was blocked with 1 M ethanolamine at pH 8.5, followed by regeneration using 50 mM NaOH. The interaction experiments were performed using running buffer containing 10 mM HEPES pH 7.5, 150 mM NaCl, and 1 mM Tris(2-carboxyethyl)phosphine and 0.1% Tween-20. Binding experiments were carried out using a Gβ_1_γ_1_ concentration range of 0.6–312 nM in running buffer at a flow rate of 30 µL min^−1^. The association and dissociation kinetics for Gβ_1_γ_1_ were monitored for 140 and 300 s, respectively. SPR data processing and analysis were performed using Biacore T100 Evaluation Software (GE, version 2.0.3). For kinetic analyses, data were locally fit to a 1:1 Langmuir model to obtain on-rate and off-rate constants.

### G protein extraction from mouse brain

G proteins were extracted from mouse brain using a protocol described elsewhere with a few modifications^69^. Brains from 32 C57BL/6 J mice (Jackson laboratory) were thawed in 50 ml buffer containing 20 mM Tris-HCl, pH 8.0, 1 mM EDTA, 1 mM DTT, 3 mM MgCl_2_ and halt protease inhibitor cocktail (ThermoFisher). Thawed brains were homogenized and centrifuged at 39,000 *g* for 20 min at 4 °C. Membrane pellets were homogenized in 50 ml buffer A containing 20 mM Tris-HCl, pH 8.0, 1 mM EDTA, 1 mM DTT and halt protease inhibitor cocktail and washed twice after adding buffer A by centrifugation at 39,000 *g* for 20 min. Pellets then were resuspended and washed by centrifugation in buffer A supplemented with 0.1 M NaCl and 0.1% (w/v) sodium cholate. Next, membranes were homogenized and solubilized in buffer A containing 2% (w/v) sodium cholate for 2 h at 4 °C. The clear supernatant obtained after centrifugation at 186,000 *g* was loaded onto a DEAE Sepharose column equilibrated with buffer A containing 20 mM NaCl and 0.2% sodium cholate. Bound proteins were eluted with a linear gradient of NaCl (0–500 mM) in buffer A with 0.2% sodium cholate. The eluate was desalted, supplemented and incubated with a two molar excess of either Nb5 or Nb17 for 1 h and then subjected to immobilized-Ni^2+^ affinity chromatography. The Ni^2+^-NTA resin was equilibrated with buffer A containing 0.1 M NaCl, 0.2% sodium cholate and 5 mM imidazole and this was added to the eluate obtained during DEAE Sepharose purification. Following 1 h of incubation, the resin was washed with 50 resin volumes of buffer A containing 0.1 M NaCl, 0.2% sodium cholate and 5 mM imidazole. The Gβγ-Nb5 complex then was eluted with buffer A containing 0.1 M NaCl, 0.1% sodium cholate and 300 mM imidazole. The Ni^2+^-NTA purified Gβγ-Nb5 complex was desalted and loaded onto 500 μl of Talon resin packed into a Pierce™ disposable column (ThermoFisher) equilibrated with buffer A containing 0.1 M NaCl, 0.1% sodium cholate, and 5 mM imidazole. The Talon resin was washed with 50 resin volumes of equilibration buffer and the purified Gβγ-Nb5 complex was eluted with buffer A containing 0.1 M NaCl, 0.1% sodium cholate and 300 mM imidazole.

### In-gel protein digestion of Gβ subtypes

The excised SDS-PAGE band was destained in a solution containing 50% (v/v) ethanol with 5% (v/v) acetic acid in water. Next, the gel band was treated with acetonitrile and 5 μM DTT followed by its alkylation with iodoacetamide. Complete in-gel protein digestion was achieved by treatment with 50 ng of trypsin and chymotrypsin proteases prepared in 50 mM ammonium bicarbonate for 16 h at room temperature. Peptides were extracted by washing the gel twice with 30 μL of 50% (v/v) acetonitrile with 5% (v/v) formic acid in water. Extracts were combined and evaporated to <10 μL in a Speedvac and then resuspended in 1% (v/v) acetic acid to achieve a final volume of 30 μL. Next, the sample was loaded on to a Dionex 15 cm × 75 μm id Acclaim Pepmap C18, 2 μm, 100 Å reversed phase capillary chromatography column attached to a Finnigan LTQ-Obitrap Elite hybrid mass spectrometer system. Peptides were eluted with a gradient of 0.1% (v/v) formic acid in acetonitrile at a flow rate of 0.3 μL min^−1^ and these were injected into the electrospray ionization source of the mass spectrometer on-line. The nano-electrospray ion source was operated at 1.9 kV. The digest was analyzed using the data-dependent multitask capability of the instrument acquiring full scan mass spectra to determine peptide molecular weights and product ion spectra to obtain the amino acid sequence in successive instrument scans. Data were analyzed by using all CID spectra collected in the experiment to search whole mouse UniProtKB databases with the search program Sequest. Search parameters for peptide identification were as follows: minimum precursor mass, 350 Da; maximum precursor mass, 5000 Da; maximum missed cleavage sites, 2 for trypsin and 6 for chymotrypsin digestion; minimum peptide length, 6; maximum peptide length, 144; precursor mass tolerance, 10 ppm; fragment mass tolerance, 0.8 Da; static modification, carbamidomethylation; dynamic modification, oxidation for trypsin and oxidation and phosphorylation for chymotrypsin digestion; maximum dynamic modifications per peptide, 4; and maximum Delta Cn (degree of match between the scores of the possible peptide spectral matches), 0.05. Sequest searches were also performed against five Gβ subtypes using the same parameters to confirm the identities of the peptides and the sequence coverage. Data are available via ProteomeXchange with identifier PXD009503.

### Gβ subunit selectivity of Nb5

HEK293T/17 cells (American Type Culture Collection, Manassas, VA) were chosen for this analysis because of their high transfectability^70^. Cells were grown in culture medium (Dulbecco’s modified Eagle’s medium) supplemented with 10% fetal bovine serum, MEM non-essential amino acids (Life Technologies), 1 mM sodium pyruvate, and antibiotics (100 units ml^−1^ penicillin and 100 μg ml^−1^ streptomycin) at 37 °C in a humidified incubator containing 5% CO_2_. For transfection, 6-cm culture dishes were coated during incubation for 10 min at 37 °C with 2.5 ml of Matrigel solution (approximately 10 μg ml^−1^ growth factor-reduced Matrigel (BD Biosciences) in culture medium). Cells were seeded into the 6-cm dishes containing Matrigel solution at a density of 3.5 × 10^6^ cells/dish. After 4 h, expression constructs (total 10 μg/dish) were transfected into the cells using PLUS (10 μl/dish) and Lipofectamine LTX (12 μl/dish) reagents. Gα_oA_, Venus 1–155 Gβ, Venus-156–239-Gγ2, masGRK3ct-Nluc, and nanobody constructs were used at a 2:1:1:1:12 ratio (ratio 1 = 0.42 μg of plasmid DNA). Empty vector pcDNA3.1(+) was employed to normalize the amounts of transfected DNA.

Cellular measurements of BRET between Venus-Gβγ and masGRK3ct-Nluc-HA were performed to examine the Gβ selectivity of Nb5 in living cells. Sixteen to twenty-four hours post-transfection, HEK293T/17 cells were washed once with BRET buffer (PBS containing 0.5 mM MgCl_2_ and 0.1% glucose) and detached by gentle pipetting with BRET buffer. Cells were harvested by centrifugation at 500 *g* for 5 min and resuspended in BRET buffer. Approximately 50,000–100,000 cells per well were distributed in 96-well flat-bottomed white microplates (Greiner Bio-One). The Nluc substrate, furimazine, purchased from Promega, was used according to the manufacturer’s instructions. BRET measurements were made with a micro plate reader (POLARstar Omega; BMG Labtech). All measurements were performed at room temperature. The BRET ratio was determined by calculating the ratio of the light emitted by the Venus-Gβγ (535 nm with a 30 nm band path width) over the light emitted by the masGRK3ct-Nluc-HA (475 nm with a 30 nm band path width). BRET assays for real-time monitoring of cAMP and Ca^2+^ were performed in the same manner as described above. At approximately 16–24 h post-transfection, cells were stimulated with either 100 μM acetylcholine (ACh) or dopamine. Full blots are shown in Supplementary Figure 4a.

### Stereotaxic injection

All animal procedures were performed with the approval of the Institutional Animal Care and Use Committee at Case Western Reserve University. To allow the expression of G protein-coupled inwardly rectifying potassium channels (GIRKs, Kir3.2) in striatal MSNs, wild type (WT) C57BL6 mice (Jackson Laboratory) were injected with adeno-associated virus encoding mGIRK2 (AAV.GIRK2, TdTomato) into the dorsal striatum at postnatal day 21. Stereotaxic injections were performed while mice were under anesthesia. AAV.GIRK2, 300 nL, was injected into one hemisphere of the dorsal striatum. The injection coordinates were: AP + 1.15 mm, ML + 1.825 mm, DV −3.325 mm (relative to the bregma). Animals were allowed to recover for ~3 weeks after surgery.

### Brain slice preparation

Three weeks after surgery, mice were euthanized and coronal brain slices containing the striatum were made in ice cold cutting solution containing 75 mM NaCl, 2.5 mM KCl, 6 mM MgCl_2_, 0.1 mM CaCl_2_, 1.2 mM NaH_2_PO_4_, 25 mM NaHCO_3_, 2.5 mM D-glucose, and 50 mM sucrose bubbled with 95% O_2_ and 5% CO_2_. Slices were incubated for 1 h at 32 °C in artificial cerebrospinal fluid (ACSF) containing: 126 mM NaCl, 2.5 mM KCl, 1.2 mM MgCl_2_, 2.4 mM CaCl_2_, 1.2 mM NaH_2_PO_4_, 21.4 mM NaHCO_3_, 11.1 mM D-glucose and 10 μM MK-801 bubbled with 95% O_2_ and 5% CO_2_. Slices then were transferred to a recording chamber and perfused with ACSF at 32 °C containing picrotoxin (100 μM) and DNQX (10 μM).

### Electrophysiological recordings

Striatal neurons were visualized with an Olympus BX51 fluorescence microscope, and GIRK2 expressing MSNs were identified by the presence of tdTomato. Electrical stimulation was applied with a monopolar extracellular stimulating electrode filled with ACSF. A single stimulation (1 ms, 20–35 μA) was used to evoke the release of neurotransmitters in the striatum. Whole-cell voltage clamp recordings were performed with an Axopatch 200B amplifier. Patch pipettes were filled with an internal solution containing 135 mM D-gluconate(K), 20 mM NaCl, 1.5 mM MgCl_2_, 10 mM HEPES(K), pH 7.4, 10 mM BAPTA-tetrapotassium, 1 mg ml^−1^ ATP, 0.1 mg ml^−1^ GTP, 1.5 mg ml^−1^ phosphocreatine, and 10 μM of either Nb5 or Nb17 (275 mOsm). Recordings were acquired with Axograph X (Axograph Scientific) at 10 kHz and filtered to 2 kHz for analysis. Neurons were held at −60 mV. No series resistance compensation was used, and neurons were discarded if the series resistance exceeded 15 MΩ.

### Cell culture and transfection

A Chinese hamster ovary cell-apelin receptor (CHO-APJ) stable cell line (American Type Culture Collection, Manassas, VA) was established in the lab. Briefly, human apelin receptor (APJ) cDNA was synthesized (GenScript, Piscataway, NJ) and inserted into pLNCX2 (catalog no. 631503, Clontech, Inc.). Stable cell lines were generated by viral infection and G418 selection at a concentration of 800 µg ml^−1^. For expression of Nb5 and Nb17 in mammalian cells, cDNAs of the nanobodies were sub-cloned into pcDNA3.1 (+) (Catalog no: V79020, Thermo Fisher Scientific, Inc) with a Flag tag at the C-terminus. CHO-APJ cells were transiently transfected with lipofectamine 2000 (Invitrogen, Carlsbad, CA). All cells were incubated overnight at 37 °C in serum-free medium containing 1% BSA before treatment with 1 μm apelin or a vehicle control. For immunoblotting, proteins on the blots were detected with the indicated antibodies using the Odyssey Fc Imaging System (LI-COR Biosciences, Lincoln, NE). The intensity of the bands was quantified using Image Studio Lite 5.2 software. Full blots are reported in Supplementary Figure 4b.

### cAMP assay

CHO-APJ cells expressing Nb5 or Nb17 were plated onto a 96-well plate at 80,000 cells/well and incubated at 37 °C overnight in 5% CO_2_. On the day of the cAMP assay, cells were treated with IBMX at a final concentration of 750 µM for 20 min at room temperature. Cells then were treated with forskolin (20 µM), together with apelin-13 at various concentrations for 10 min. After these reactions, cells were lysed and their intracellular cAMP levels were measured according to the procedures of the CatchPoint Cyclic-AMP Fluorescent Assay Kit (Molecular Devices, Sunnyvale, CA USA). Fluorescence with excitation/emission at 530/590 nm was read with a Flexstation3 plate reader (Molecular Devices).

### Statistics

Differential HDX of Gβ_1_γ_1_ alone and the Gβ_1_γ_1_-Nb5 complex was performed on three independent repeats and statistical significance was determined with two-tailed student’s *t*-test where *P* values of <0.01 were considered significant. The BRET assays were performed on six replicates where one-way ANOVA with Tukey’s post hoc multiple comparison test was applied relative to the Gβ_1_γ_2_/GRK3ct control. The *P* values of <0.001 were considered significant and the results were expressed as mean ± SEM. Nb5 electrophysiological recordings were obtained from nine striatum neurons for the measurement of D2R-IPSCs and eight neurons for the measurements of M4R-IPSCs. Nb17 electrophysiological recordings were obtained from five striatum neurons for the measurement of D2R-IPSCs. The amplitudes obtained from individual recordings were then averaged and the significance (*P* < 0.05) was determined with two-tailed student’s *t*-test. The real-time monitoring of Ca^2+^ was performed on six replicates where two-way ANOVA with Tukey’s post hoc multiple comparison test was applied. The BRET-based cAMP assay was performed on three independent experiments where two-way ANOVA with Tukey’s post hoc multiple comparison test was applied.

### Data availability statement

Data supporting the findings of this manuscript are available from the corresponding author upon reasonable request. Coordinates and structure factor amplitudes of the Gβ_1_γ_1_-Nb5 complex are deposited in the PDB (PDB accession: 6B20). The mass spectrometry proteomics data have been deposited to the ProteomeXchange Consortium via the PRIDE partner repository with the dataset identifier PXD009503.

## Electronic supplementary material

Supplementary Information

Peer Review File

## References

[1] Palczewski K (2006). G protein-coupled receptor rhodopsin. Annu. Rev. Biochem..

[2] Rosenbaum DM, Rasmussen SG, Kobilka BK (2009). The structure and function of G-protein-coupled receptors. Nature.

[3] Oldham WM, Hamm HE (2008). Heterotrimeric G protein activation by G-protein-coupled receptors. Nat. Rev. Mol. Cell Biol..

[4] Bondar A, Lazar J (2014). Dissociated GalphaGTP and Gbetagamma protein subunits are the major activated form of heterotrimeric Gi/o proteins. J. Biol. Chem..

[5] Santos R (2017). A comprehensive map of molecular drug targets. Nat. Rev. Drug. Discov..

[6] Kenakin T (2002). Efficacy at G-protein-coupled receptors. Nat. Rev. Drug. Discov..

[7] Violin JD, Crombie AL, Soergel DG, Lark MW (2014). Biased ligands at G-protein-coupled receptors: promise and progress. Trends Pharmacol. Sci..

[8] Leeson PD, Springthorpe B (2007). The influence of drug-like concepts on decision-making in medicinal chemistry. Nat. Rev. Drug. Discov..

[9] Hutchings CJ, Koglin M, Marshall FH (2010). Therapeutic antibodies directed at G protein-coupled receptors. Mabs.

[10] Pardon E (2014). A general protocol for the generation of Nanobodies for structural biology. Nat. Protoc..

[11] De Genst E (2006). Molecular basis for the preferential cleft recognition by dromedary heavy-chain antibodies. Proc. Natl Acad. Sci. USA.

[12] Jahnichen S (2010). CXCR4 nanobodies (VHH-based single variable domains) potently inhibit chemotaxis and HIV-1 replication and mobilize stem cells. Proc. Natl Acad. Sci. USA.

[13] Bradley ME (2015). Potent and efficacious inhibition of CXCR2 signaling by biparatopic nanobodies combining two distinct modes of action. Mol. Pharmacol..

[14] Griffiths K (2013). Shark variable new antigen receptor (VNAR) single domain antibody fragments: stability and diagnostic applications. Antibodies.

[15] Griffiths K (2016). i-bodies, human single domain antibodies that antagonize chemokine receptor CXCR4. J. Biol. Chem..

[16] Ghosh E (2017). A synthetic intrabody-based selective and generic inhibitor of GPCR endocytosis. Nat. Nanotechnol..

[17] Dorsam RT, Gutkind JS (2007). G-protein-coupled receptors and cancer. Nat. Rev. Cancer.

[18] Brock, C., et al. Roles of G beta gamma in membrane recruitment and activation of p110 gamma/p101 phosphoinositide 3-kinase gamma. *J. Cell Biol*. **160**, 89–99 (2003). 10.1083/jcb.200210115 PMC2172741 12507995

[19] Tang WJ, Gilman AG (1991). Type-specific regulation of adenylyl cyclase by G protein beta gamma subunits. Science.

[20] Inglese J, Koch WJ, Touhara K, Lefkowitz RJ (1995). G beta gamma interactions with PH domains and Ras-MAPK signaling pathways. Trends Biochem. Sci..

[21] Loew A, Ho YK, Blundell T, Bax B (1998). Phosducin induces a structural change in transducin beta gamma. Structure.

[22] Lin Y, Smrcka AV (2011). Understanding molecular recognition by G protein betagamma subunits on the path to pharmacological targeting. Mol. Pharmacol..

[23] Fahmy K, Sakmar TP (1993). Regulation of the rhodopsin-transducin interaction by a highly conserved carboxylic acid group. Biochemistry.

[24] Carman CV (2000). Mutational analysis of Gbetagamma and phospholipid interaction with G protein-coupled receptor kinase 2. J. Biol. Chem..

[25] Bauer PH (1998). Interactions of phosducin with the subunits of G-proteins. Binding to the alpha as well as the betagamma subunits. J. Biol. Chem..

[26] Rasmussen SG (2011). Crystal structure of the beta2 adrenergic receptor-Gs protein complex. Nature.

[27] Lodowski DT, Pitcher JA, Capel WD, Lefkowitz RJ, Tesmer JJ (2003). Keeping G proteins at bay: a complex between G protein-coupled receptor kinase 2 and Gbetagamma. Science.

[28] Krissinel E, Henrick K (2007). Inference of macromolecular assemblies from crystalline state. J. Mol. Biol..

[29] Lawrence MC, Colman PM (1993). Shape complementarity at protein/protein interfaces. J. Mol. Biol..

[30] Smrcka AV (2010). NMR analysis of G-protein betagamma subunit complexes reveals a dynamic G(alpha)-Gbetagamma subunit interface and multiple protein recognition modes. Proc. Natl Acad. Sci. USA.

[31] Sarvazyan NA, Remmers AE, Neubig RR (1998). Determinants of gi1alpha and beta gamma binding. Measuring high affinity interactions in a lipid environment using flow cytometry. J. Biol. Chem..

[32] Wang J, Golebiewska U, Scarlata S (2009). A self-scaffolding model for G protein signaling. J. Mol. Biol..

[33] Masuho I (2015). Distinct profiles of functional discrimination among G proteins determine the actions of G protein-coupled receptors. Sci. Signal..

[34] Papermaster DS (1982). Preparation of retinal rod outer segments. Methods Enzymol..

[35] Mamaligas AA, Ford CP (2016). Spontaneous synaptic activation of muscarinic receptors by striatal cholinergic neuron firing. Neuron.

[36] Olianas MC, Onali P (1999). Mediation by G protein betagamma subunits of the opioid stimulation of adenylyl cyclase activity in rat olfactory bulb. Biochem. Pharmacol..

[37] Defer N, Best-Belpomme M, Hanoune J (2000). Tissue specificity and physiological relevance of various isoforms of adenylyl cyclase. Am. J. Physiol. Ren. Physiol..

[38] Perez C (2017). Structural basis of inhibition of lipid-linked oligosaccharide flippase PglK by a conformational nanobody. Sci. Rep..

[39] Geertsma ER (2015). Structure of a prokaryotic fumarate transporter reveals the architecture of the SLC26 family. Nat. Struct. Mol. Biol..

[40] Bonacci TM (2006). Differential targeting of Gbetagamma-subunit signaling with small molecules. Science.

[41] Hwang JI, Choi S, Fraser ID, Chang MS, Simon MI (2005). Silencing the expression of multiple Gbeta-subunits eliminates signaling mediated by all four families of G proteins. Proc. Natl Acad. Sci. USA.

[42] Koch WJ, Hawes BE, Inglese J, Luttrell LM, Lefkowitz RJ (1994). Cellular expression of the carboxyl terminus of a G protein-coupled receptor kinase attenuates G beta gamma-mediated signaling. J. Biol. Chem..

[43] Goubaeva F (2003). Stimulation of cellular signaling and G protein subunit dissociation by G protein betagamma subunit-binding peptides. J. Biol. Chem..

[44] Scott JK (2001). Evidence that a protein-protein interaction ‘hot spot’ on heterotrimeric G protein betagamma subunits is used for recognition of a subclass of effectors. EMBO J..

[45] Bonacci TM, Ghosh M, Malik S, Smrcka AV (2005). Regulatory interactions between the amino terminus of G-protein betagamma subunits and the catalytic domain of phospholipase Cbeta2. J. Biol. Chem..

[46] Koch WJ, Hawes BE, Allen LF, Lefkowitz RJ (1994). Direct evidence that Gi-coupled receptor stimulation of mitogen-activated protein kinase is mediated by G beta gamma activation of p21ras. Proc. Natl Acad. Sci. USA.

[47] Neptune ER, Bourne HR (1997). Receptors induce chemotaxis by releasing the betagamma subunit of Gi, not by activating Gq or Gs. Proc. Natl Acad. Sci. USA.

[48] Premont RT (1996). Identification and characterization of a widely expressed form of adenylyl cyclase. J. Biol. Chem..

[49] Bayewitch ML (1998). Differential modulation of adenylyl cyclases I and II by various G beta subunits. J. Biol. Chem..

[50] Koide S (2009). Engineering of recombinant crystallization chaperones. Curr. Opin. Struct. Biol..

[51] Thueng-in K (2012). Cell penetrable humanized-VH/V(H)H that inhibit RNA dependent RNA polymerase (NS5B) of HCV. PLoS ONE.

[52] Abulrob A, Sprong H, Van Bergen en Henegouwen P, Stanimirovic D (2005). The blood-brain barrier transmigrating single domain antibody: mechanisms of transport and antigenic epitopes in human brain endothelial cells. J. Neurochem..

[53] Pardridge WM (2015). Targeted delivery of protein and gene medicines through the blood-brain barrier. Clin. Pharmacol. Ther..

[54] Hollins B, Kuravi S, Digby GJ, Lambert NA (2009). The c-terminus of GRK3 indicates rapid dissociation of G protein heterotrimers. Cell Signal..

[55] Zell R, Fritz HJ (1987). DNA mismatch-repair in Escherichia coli counteracting the hydrolytic deamination of 5-methyl-cytosine residues. EMBO J..

[56] Baker BY (2015). Crystallization of proteins from crude bovine rod outer segments. Methods Enzymol..

[57] Hofmann L, Gulati S, Sears A, Stewart PL, Palczewski K (2016). An effective thiol-reactive probe for differential scanning fluorimetry with a standard real-time polymerase chain reaction device. Anal. Biochem..

[58] Gulati S (2017). Photocyclic behavior of rhodopsin induced by an atypical isomerization mechanism. Proc. Natl Acad. Sci..

[59] Heck M, Hofmann KP (2001). Maximal rate and nucleotide dependence of rhodopsin-catalyzed transducin activation: initial rate analysis based on a double displacement mechanism. J. Biol. Chem..

[60] Kabsch W (2010). Xds. Acta Crystallogr. D. Biol. Crystallogr..

[61] Nguyen VS (2017). Type VI secretion TssK baseplate protein exhibits structural similarity with phage receptor-binding proteins and evolved to bind the membrane complex. Nat. Microbiol.

[62] Collaborative Computational Project, N. (1994). The CCP4 suite: programs for protein crystallography. Acta Crystallogr. D. Biol. Crystallogr..

[63] McCoy AJ (2007). Solving structures of protein complexes by molecular replacement with Phaser. Acta Crystallogr. D. Biol. Crystallogr..

[64] Emsley P, Cowtan K (2004). Coot: model-building tools for molecular graphics. Acta Crystallogr. D. Biol. Crystallogr..

[65] Davis IW (2007). MolProbity: all-atom contacts and structure validation for proteins and nucleic acids. Nucleic Acids Res..

[66] Berman H, Henrick K, Nakamura H (2003). Announcing the worldwide Protein Data Bank. Nat. Struct. Biol..

[67] Orban T, Tsybovsky Y (2015). Probing conformational changes in rhodopsin using hydrogen-deuterium exchange coupled to mass spectrometry. Methods Mol. Biol..

[68] Guttman M, Weis DD, Engen JR, Lee KK (2013). Analysis of overlapped and noisy hydrogen/deuterium exchange mass spectra. J. Am. Soc. Mass. Spectrom..

[69] Katada T, Oinuma M, Ui M (1986). Two guanine nucleotide-binding proteins in rat brain serving as the specific substrate of islet-activating protein, pertussis toxin. Interaction of the alpha-subunits with beta gamma-subunits in development of their biological activities. J. Biol. Chem..

[70] Pear WS, Nolan GP, Scott ML, Baltimore D (1993). Production of high-titer helper-free retroviruses by transient transfection. Proc. Natl Acad. Sci. USA.

