# Supplementary material for: Targeting G protein-coupled receptor signaling at the G protein level with a selective nanobody inhibitor
Source: Nat Commun. 2018 May 18;9:1996. doi: 10.1038/s41467-018-04432-0 (PMC5959942; doi:10.1038/s41467-018-04432-0)
Supplement: Supplementary file 1 — Supplementary Information [file 41467_2018_4432_MOESM1_ESM.pdf]

Supplementary Information

Targeting G protein-coupled receptor signaling at the G protein level with a selective nanobody inhibitor

Gulati et al.

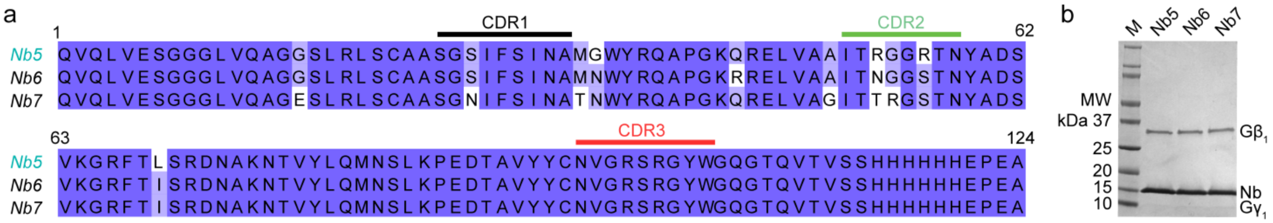

Supplementary Figure 1: Nanobodies directed towards G$\beta_1\gamma_1$ dimer. (**a**) Multiple sequence alignment of the three members of the Nb5 family, Nb5, Nb6 and Nb7 having an identical complementarity determining region 3 (CDR3). Complementarity determining regions 1, 2 and 3 are marked with black, green, and red lines, respectively. (**b**) Coomassie stained SDS-polyacrylamide gel showing the ability of Nb5, Nb6 and Nb7 to selectively trap the G$\beta_1\gamma_1$ dimer.

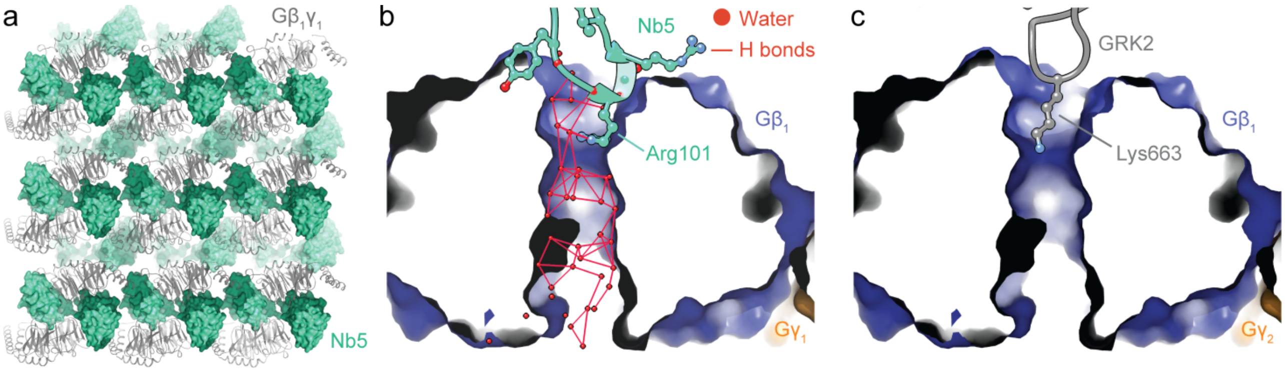

Supplementary Figure 2: Interaction of Nb5 with the G$\beta_1\gamma_1$ dimer. G$\beta_1\gamma_1$-Nb5 was crystallized in space group, $P2_1$, where most crystal contacts were mediated through Nb5 within the aqueous layers. (**a**) Top view of the G$\beta_1\gamma_1$-Nb5 complex crystal lattice in a direction perpendicular to the long C-axis. Each asymmetric unit is composed of two G$\beta_1\gamma_1$-Nb5 molecules that form crystal contacts with neighboring molecules. (**b**) Arg-101 in the CDR3 region of Nb5 serves as a key that locks the Gβ_1-propeller and forms an intricate hydrogen-bonding network with water molecules in the Gβ_1-propeller cavity. (**c**) Lys-663 in the C-terminal loop of G Protein-Coupled Receptor Kinase 2 (GRK2) forms a similar lock and key interaction in the G$\beta_1\gamma_2$-GRK2 complex (PDB accession: 1OMW[1]).

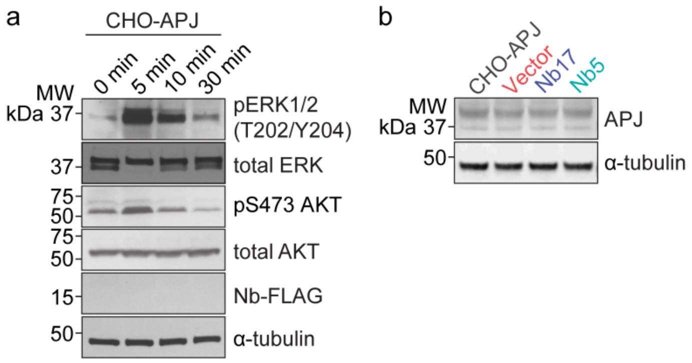

Supplementary Figure 3: Effect of Nb5 on GPCR-mediated Gβγ signaling. (**a**) Wild-type CHO-APJ cells were treated with 1 μM apelin over 0-30 min at 37°C, and the optimum time point of 5 min was selected for further experiments based on the phosphorylation levels of both ERK1/2 and AKT. (**b**) Western blot analysis showed similar expression levels of the apelin receptor in parental CHO-APJ cells, CHO-APJ-vector cells, CHO-APJ-Nb5 and CHO-APJ-Nb17 cells.

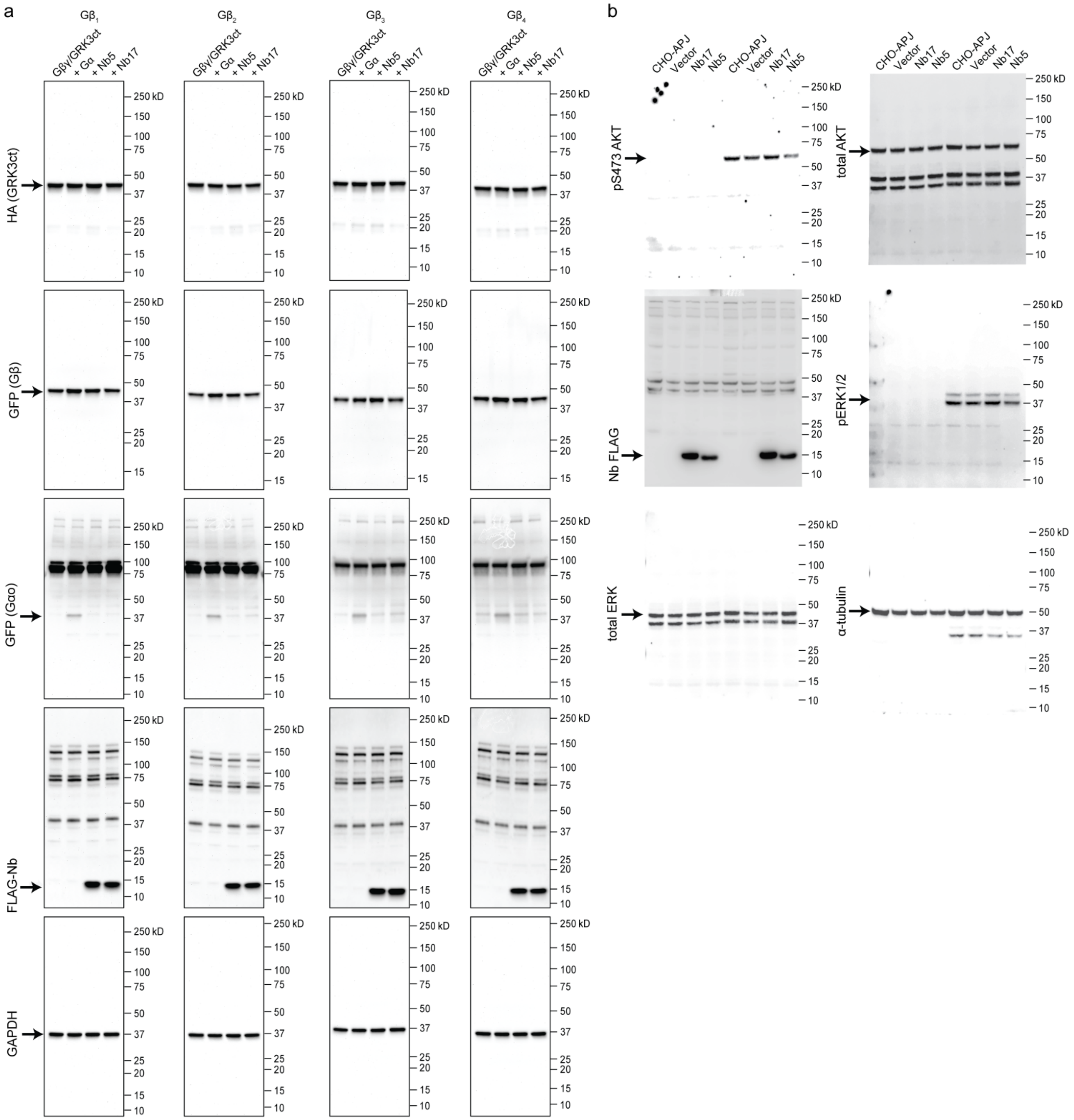

Supplementary Figure 4: Full Western blots. (**a**) Western blots showing the quantification of the expression levels of Gβ1-4, GRK3, Gα, Nb5, and Nb17. (**b**) Western blots demonstrating reduced phosphorylation of both pERK1/2 and pAKT in CHO-APJ-Nb5 cells as compared to the CHO-APJ, CHO-APJ-vector, and CHO-APJ-Nb17 cells.

Supplementary Table 1: Sequences of $G\beta_1\gamma_1$ peptide fragments showing normalized deuterium uptake for the $G\beta_1\gamma_1$ complex alone and the $G\beta_1\gamma_1$ complex bound to Nb5. Column 1 shows the peptide sequences from the $G\beta_1\gamma_1$ primary amino acid sequence. The numbers shown in brackets indicate the positions of peptides in the primary protein sequence. Column 2 reveals the mass over charge (m/z) ratio of the ion used to identify the peptide based on its MS/MS spectrum. Column 3 displays the charge of the ion from Column 2. Column 4 indicates the maximum number of theoretically H/D exchangeable sites in the peptide fragment (max = number of non-proline peptide bonds in the peptide fragment). Column 5 shows the deuterium uptake normalized to 80% of the theoretical maximum exchangeable sites. The 80% normalization reflects the dilution used during the sample preparation in D_2O (see *Methods*). Column 6 reports the retention time (in min) of the listed peptides. Columns 7 and 8 indicate the percentage deuterium uptake of the exchangeable sites for the $G\beta_1\gamma_1$ complex and Nb5-bound $G\beta_1\gamma_1$ complex, respectively. Column 9 shows the difference of deuterium uptake in $G\beta_1\gamma_1$ with and without Nb5. Column 9 is colored according to the percentage change in the deuterium uptake observed between $G\beta_1\gamma_1$ complex and Nb5-bound $G\beta_1\gamma_1$ complex (<-5%, *light purple;* -5%<x<-10%, *purple;* <+5%, *light greencyan*; +5%<x<+10%, *greencyan*).

Sequence	m/z	z	#Sites	#Sites (80%)	RT (min)	$G\beta_1\gamma_1$ (%)	$G\beta_1\gamma_1$-Nb5 (%)	∆HDX (%)
DQLRQEAEQLKNQIRDARKACADATL [5:30]	996.08	+3	25	20	11.81-14.42	20.36±0.38	19.21±1.11	N.S.
SQITNNIDPVGRIQM [31:45]	843.89	+2	13	10.4	12.19-12.94	50.44±0.72	47.56±4.51	N.S.
YAMHWGTDSRL [59:69]	669.25<br>446.61	+2<br>+3	10	8	14.14-14.99	17.46±2.24	17.19±1.69	N.S.
LVSASQDGKL [70:79]	1017.75<br>509.59	+1<br>+2	9	7.2	11.30	42.17±3.62	39.93±1.14	N.S.
IIWDSYTTNKVHAIPLRSSW [80:99]	1194.41<br>796.66	+2<br>+3	18	14.4	11.6-11.8	25.75±0.81	19.68±0.68	~5 %
CAYAPSGNY [103:111]	945.51	+1	7	5.6	10.49-11.50	23.65±4.36	20.59±2.78	N.S.
YVACGGLD [111:118]	797.50	+1	7	5.6	11.58	30.64±0.19	20.48±0.86	~10 %
LAGHTGYLSC [139:148]	511.51	+2	9	7.2	11.28	10.33±0.56<br>6.79±1.91	N.A<br>6.61±3.85	N.S.
IVTSSGDTTCAL [157:168]	1168.60	+1	11	8.8	13.85	53.15±2.78	59.40±7.66	N.S.
WDIETGQQTTTF [169:180]	714.11<br>714.67	+2<br>+2	11	8.8	11.40-11.74	5.53±0.73	7.74±4.96	N.S.
WDIETGQQTTTFTGHTGDVMSL [169:190]	1213.40	+2	21	16.8	12.43-14.4	22.76±0.52	23.66±1.40	N.S.
LSLAPDTRL [190:198]	985.67	+1	7	5.6	11.82-11.97	29.17±1.30	29.56±2.60	N.S.
FVSGACDASAKL [199:210]	584.75<br>1169.72	+2<br>+1	11	8.8	13.73-16.21	20.05±1.07	21.17±2.34	N.S.
WDVREGM [211:217]	446.72<br>892.60	+2<br>+1	6	4.8	12.12-12.55	50.68±4.49	51.11±4.38	N.S.

CRQTFTGHESDINA [218:231]	789.90	+2	13	10.4	11.14	19.17±1.68	16.90±1.21	N.S.
ICFFPNGNA [232:240]	982.58	+1	7	5.6	9.87-12.13	40.88±2.74	41.19±0.13	N.S.
FATGSDDATCRL [241:252]	628.77	+2	11	8.8	10.99-11.61	14.31±2.81	16.03±0.10	N.S.
FDLRADQEL [253:261]	553.97	+2	8	6.4	12.14	16.28±3.27	15.82±3.39	N.S.
FDLRADQELMT [253:263]	670.27	+2	10	8	11.75-12.74	18.16±1.06	16.54±2.02	N.S.
YSHDNIIC [264:271]	964.54<br>482.95	+1<br>+2	7	5.6	11.22	19.11±1.03	17.30±3.03	N.S.
GITSVSF [272:278]	710.41	+1	6	4.8	12.37	10.85±0.23	11.77±0.99	N.S.
SKSGRLLLAG [279:288]	501.63	+2	9	7.2	11.32	14.06±3.44	13.11±0.35	N.S.
LLAGYDDF [285:292]	913.50	+1	7	5.6	10.53-12.64	43.53±1.73	41.40±2.46	N.S.
NCNVWDALKADRAGVL [293:308]	873.23<br>582.83	+2<br>+3	15	12	12.25-12.85	19.05±2.76	17.43±1.88	N.S.
AGHDNRVSCL [309:318]	536.70	+2	9	7.2	9.93-10.70	10.87±1.41	16.41±0.63	~6 %
GVTDDGMA [319:326]	765.37	+1	7	5.6	10.22-10.86	11.09±1.34	13.60±0.49	~2 %
VATGSWDSF [327:335]	969.50	+1	8	6.4	12.28-12.53	13.63±0.59	13.23±0.68	N.S.
LKIWN [336:340]	673.53<br>337.22	+1<br>+2	4	3.2	10.96-12.08	0.22±0.44	0.81±0.58	N.S.
PVINIEDL [342:348]	912.68	+1	6	4.8	12.96-13.11	51.05±1.67	47.69±2.61	N.S.
LTEKDKLKMEVDQL [349:362]	845.62<br>564.23	+2<br>+3	13	10.4	11.15	71.37±0.84	67.88±4.60	N.S.
EVDQLKKEVTL [358:368]	651.60<br>434.87	+2<br>+3	10	8	11.53-12.01	21.97±1.36	20.21±3.15	N.S.
ERMLVSKC [369:376]	965.72	+1	7	5.6	10.44-11.55	29.34±1.42	27.03±3.37	N.S.
YVEERSGEDPLVKGIPEDKNPFKELKG [383:409]	1025.61	+3	23	18.4	11.54-12.10	31.25±0.67	30.18±0.84	N.S.

Supplementary Table 2: Diffraction data collection and structural refinement statistics for the G$\beta_1\gamma_1$-Nb5 complex.

	G$\beta_1\gamma_1$-Nb5 complex
**Data collection**	
Beamline	NE-CAT 24-ID-E
Space group	$P2_1$
Cell dimensions	
a, *b*, *c* (Å)	72.66, 77.23, 101.66
α, β, γ (°)	90.0, 90.0, 109.80
Resolution (Å) †#	50.00-2.34 (2.40-2.34)
R_{meas}I (%)†	22 (177.0)
$CC_{1/2}$ (%) †	99.1 (50.7)
<I/σ>†	6.28 (1.05)
Completeness (%)	99.6 (99.8)
Redundancy	4.31 (4.43)
Wilson *B*-factor (Å^2)	48
**Refinement**	
Resolution (Å)	2.34
No. of unique reflections	42434 (3268)
R_{work} / R_{free} (%)	20/24.9
No. atoms	
Protein	7893
Ligand/ion	12
Water	396
B-factors	
Overall	52.9
Atoms of protein	53.4
Atoms of solvent water	43.8
R.m.s. deviations	
Bond lengths (Å)	0.0123
Bond angles (°)	1.5735
Structure evaluation	
Ramachandran favored (%)	95.43
Ramachandran outliers (%)	0.20*
Percentile	100th

†Values in parentheses are for the highest-resolution shell of data. ID, insertion device.

#Resolution bin at <I/σ> of 2.1 is 2.70-2.62 Å for comparison with the historical standards of x-ray data truncation. The resolution bin with <I/σ> of 1.05 is used for the resolution cut-off to include the intensities that are significantly above the noise level. Extending the data beyond <I/σ> values of >2 have been shown to improve structure determination in many cases with no negative impact on model building [2-4].

*Glycine Ramachandran outliers that were consistent with other G$\beta_1\gamma_1$ crystal structures.

Supplementary Table 3: List of proteins identified from the in-gel protein digestion and mass-spectrophotometry (MS) analyses of the Gβ gel band. The MS-identified peptides were searched against the full mouse proteome to eliminate false-positives. Column 1 shows the Uniprot accession IDs. Column 2 describes the proteins that were identified based on their MS/MS spectrum. Column 3 displays the sequest score which determines the quality of hits based on the number of ions in the MS/MS spectrum that match with the experimental data. Column 4 shows the percentage of the protein sequence covered by identified peptides. Column 5 shows the number of peptide sequences that are unique to a protein group and do not occur in the proteins of any other group. Column 6 reports the total number of distinct peptide sequences identified in the protein group. Column 7 displays the number of peptide spectrum matches (PSMs) that reports the total number of identified peptide spectra matched for the protein. Columns 8 report the molecular weight of the identified proteins. The Gβ subtypes are highlighted in greencyan. Data are available via ProteomeXchange with identifier PXD009503.

Accession	Description	Score	Coverage	# Unique Peptides	# Peptides	# PSMs	MW [kDa]
P62874	Guanine nucleotide-binding protein G(I)/G(S)/G(T) subunit beta-1 OS=Mus musculus GN=Gnb1 PE=1 SV=3 - [GBB1_MOUSE]	470.08	59.41	19	45	106	37.4
P62880	Guanine nucleotide-binding protein G(I)/G(S)/G(T) subunit beta-2 OS=Mus musculus GN=Gnb2 PE=1 SV=3 - [GBB2_MOUSE]	409.76	65.29	23	46	95	37.3
P29387	Guanine nucleotide-binding protein subunit beta-4 OS=Mus musculus GN=Gnb4 PE=1 SV=4 - [GBB4_MOUSE]	278.51	52.06	10	33	62	37.4
P61264	Syntaxin-1B OS=Mus musculus GN=Stx1b PE=1 SV=1 - [STX1B_MOUSE]	163.04	31.94	15	17	34	33.2
P16858	Glyceraldehyde-3-phosphate dehydrogenase OS=Mus musculus GN=Gapdh PE=1 SV=2 - [G3P_MOUSE]	81.91	42.34	14	14	27	35.8
A0A140LHL5	NAD-dependent protein deacetylase sirtuin-2 OS=Mus musculus GN=Sirt2 PE=1 SV=1 - [A0A140LHL5_MOUSE]	78.40	39.60	17	17	26	39.4
Q5D0A4	Stx1a protein (Fragment) OS=Mus musculus GN=Stx1a PE=2 SV=1 - [Q5D0A4_MOUSE]	68.41	34.84	10	12	22	32.9
P60904	DnaJ homolog subfamily C member 5 OS=Mus musculus GN=Dnajc5 PE=1 SV=1 - [DNJC5_MOUSE]	57.24	35.35	7	7	16	22.1
Q8K0S0	Phytanoyl-CoA hydroxylase-interacting protein OS=Mus musculus GN=Phyhip PE=1 SV=1 - [PHYIP_MOUSE]	43.03	23.64	8	8	14	37.5
Q9CZ42-2	Isoform 2 of ATP-dependent (S)-NAD(P)H-hydrate dehydratase OS=Mus musculus GN=Carkd - [NNRD_MOUSE]	37.99	32.55	11	11	14	32.1
Q8BMQ1	Guanine nucleotide binding protein, beta 3, isoform CRA_b OS=Mus musculus GN=Gnb3 PE=2 SV=1 - [Q8BMQ1_MOUSE]	35.34	18.24	1	8	14	32.4
P62137	Serine/threonine-protein phosphatase PP1-	28.30	21.21	2	6	8	37.5

	alpha catalytic subunit OS=Mus musculus GN=Ppp1ca PE=1 SV=1 - [PP1A_MOUSE]						
P04925	Major prion protein OS=Mus musculus GN=Prnp PE=1 SV=2 - [PRIO_MOUSE]	27.84	22.44	5	5	9	28.0
Q8R1P3	Gpm6a protein OS=Mus musculus GN=Gpm6a PE=2 SV=1 - [Q8R1P3_MOUSE]	27.07	20.97	5	5	9	29.8
P62141	Serine/threonine-protein phosphatase PP1-beta catalytic subunit OS=Mus musculus GN=Ppp1cb PE=1 SV=3 - [PP1B_MOUSE]	25.88	18.04	2	5	8	37.2
P63087	Serine/threonine-protein phosphatase PP1-gamma catalytic subunit OS=Mus musculus GN=Ppp1cc PE=1 SV=1 - [PP1G_MOUSE]	25.72	18.58	1	5	8	37.0
Q8VDN4	Coiled-coil domain-containing protein 92 OS=Mus musculus GN=Ccdc92 PE=1 SV=1 - [CCD92_MOUSE]	25.43	14.97	4	4	7	35.2
Q60829	Protein phosphatase 1 regulatory subunit 1B OS=Mus musculus GN=Ppp1r1b PE=1 SV=2 - [PPR1B_MOUSE]	24.70	27.32	3	3	8	21.8
Q8BTQ1	ELAV-like protein (Fragment) OS=Mus musculus GN=Elavl1 PE=2 SV=1 - [Q8BTQ1_MOUSE]	22.69	21.64	5	5	8	33.7
Q9CX86	Heterogeneous nuclear ribonucleoprotein A0 OS=Mus musculus GN=Hnrnpa0 PE=1 SV=1 - [ROA0_MOUSE]	19.61	19.02	4	4	7	30.5
Q6PER3	Microtubule-associated protein RP/EB family member 3 OS=Mus musculus GN=Mapre3 PE=1 SV=1 - [MARE3_MOUSE]	19.14	14.59	3	3	6	31.9
D3YYK8	Microtubule-associated protein RP/EB family member 2 (Fragment) OS=Mus musculus GN=Mapre2 PE=1 SV=1 - [D3YYK8_MOUSE]	16.21	10.77	3	3	6	29.4
P47754	F-actin-capping protein subunit alpha-2 OS=Mus musculus GN=Capza2 PE=1 SV=3 - [CAZA2_MOUSE]	12.95	16.78	4	4	5	32.9
A0A0R4J203	D-amino-acid oxidase OS=Mus musculus GN=Dao PE=1 SV=1 - [A0A0R4J203_MOUSE]	12.58	10.14	3	3	5	38.6
P19001	Keratin, type I cytoskeletal 19 OS=Mus musculus GN=Krt19 PE=1 SV=1 - [K1C19_MOUSE]	12.34	5.46	3	3	5	44.5
Q8C3D0	Putative uncharacterized protein OS=Mus musculus GN=Gpr158 PE=2 SV=1 - [Q8C3D0_MOUSE]	12.11	7.81	3	3	4	52.7
Q9CR35	Chymotrypsinogen B OS=Mus musculus GN=Ctrb1 PE=1 SV=1 - [CTRB1_MOUSE]	11.84	6.08	3	3	5	27.8
Q8R2Y0-2	Isoform 2 of Monoacylglycerol lipase ABHD6 OS=Mus musculus GN=Abhd6 - [ABHD6_MOUSE]	9.51	16.61	3	3	3	32.8
Q99J49	Tubb2a protein (Fragment) OS=Mus musculus GN=Tubb2a PE=2 SV=1 - [Q99J49_MOUSE]	9.13	12.42	3	3	3	34.0
P35803-5	Isoform 5 of Neuronal membrane glycoprotein M6-b OS=Mus musculus GN=Gpm6b - [GPM6B_MOUSE]	7.75	7.72	2	2	3	26.8

Q3U7L1	Putative uncharacterized protein (Fragment) OS=Mus musculus GN=Kcnab2 PE=2 SV=1 - [Q3U7L1_MOUSE]	6.56	6.61	1	1	2	28.8
Q9CRY7	Glycerophosphodiester phosphodiesterase domain-containing protein 1 OS=Mus musculus GN=Gdpd1 PE=1 SV=1 - [GDPD1_MOUSE]	5.90	9.55	2	2	2	35.8
O88935-1	Isoform Ib of Synapsin-1 OS=Mus musculus GN=Syn1 - [SYN1_MOUSE]	5.80	3.13	1	1	2	70.0
Q62277	Synaptophysin OS=Mus musculus GN=Syp PE=1 SV=2 - [SYPH_MOUSE]	4.89	7.32	1	1	1	34.0
Q3U844	Putative uncharacterized protein OS=Mus musculus GN=Csnk1a1 PE=2 SV=1 - [Q3U844_MOUSE]	4.80	3.69	1	1	2	37.5
Q4G0C2	Prss3 protein (Fragment) OS=Mus musculus GN=Prss3 PE=2 SV=1 - [Q4G0C2_MOUSE]	4.77	4.90	1	1	2	26.0
Q9CYK1	Tryptophan--tRNA ligase, mitochondrial OS=Mus musculus GN=Wars2 PE=1 SV=2 - [SYWM_MOUSE]	4.18	2.50	1	1	2	40.1
Q3UHG5	Tetraspanin OS=Mus musculus GN=Tspan7 PE=1 SV=1 - [Q3UHG5_MOUSE]	4.13	5.65	2	2	2	25.4
D3YVS0	Protein 1110004F10Rik OS=Mus musculus GN=1110004F10Rik PE=1 SV=1 - [D3YVS0_MOUSE]	3.85	23.40	1	1	1	10.3
P56564	Excitatory amino acid transporter 1 OS=Mus musculus GN=Slc1a3 PE=1 SV=2 - [EAA1_MOUSE]	3.33	3.68	1	1	1	59.6
A0A140LHT1	CD81 antigen (Fragment) OS=Mus musculus GN=Cd81 PE=1 SV=1 - [A0A140LHT1_MOUSE]	3.11	19.12	1	1	1	7.6
Q9CVU5	Putative uncharacterized protein (Fragment) OS=Mus musculus GN=Hnrnpl PE=2 SV=1 - [Q9CVU5_MOUSE]	3.07	19.67	1	1	1	6.7
Q62362	Testicular alpha tubulin (Fragment) OS=Mus musculus GN=Tuba-rs1 PE=2 SV=1 - [Q62362_MOUSE]	3.03	5.03	1	1	1	22.1
Q6KAM8	MFLJ00343 protein (Fragment) OS=Mus musculus GN=Flna PE=2 SV=1 - [Q6KAM8_MOUSE]	2.89	0.67	1	1	1	205.2
F7B1A4	Mitochondrial fission factor (Fragment) OS=Mus musculus GN=Mff PE=1 SV=1 - [F7B1A4_MOUSE]	2.70	9.66	1	1	1	16.0
Q9D051	Pyruvate dehydrogenase E1 component subunit beta, mitochondrial OS=Mus musculus GN=Pdhb PE=1 SV=1 - [ODPB_MOUSE]	2.47	3.62	1	1	1	38.9
D3YX62	Heme oxygenase 2 (Fragment) OS=Mus musculus GN=Hmox2 PE=1 SV=1 - [D3YX62_MOUSE]	2.08	5.26	1	1	1	26.4

Supplementary Table 4: Measurements of the apelin-induced inhibition of cAMP accumulation in CHO-APJ-Nb5, CHO-APJ-Nb5 and parental CHO-APJ cells. Column 1 shows the concentration of apelin. Column 2 reports the mean percentage intracellular cAMP/total cAMP. Column 3 displays the standard deviation associated with the mean percentage intracellular cAMP/total cAMP reported in column 2.

Concentration (nM)	NB5 (% intracellular cAMP/total cAMP)	SD
1250	35.40222779	1.07
250	38.49835167	0.883
50	35.14552504	0.755
10	44.77705617	0.964
2	70.68018383	0.928
0.4	79.7774406	0.923
0.08	82.37632058	0.685
0.016	80.607488	0.743
Concentration (nM)	NB17 (% intracellular cAMP/total cAMP)	SD
1250	55.26905291	0.36
250	56.50383218	0.585
50	55.5354657	0.04
10	59.24348369	0.434
2	73.32799974	0.131
0.4	80.08064387	0.209
0.08	81.82472759	0.422
0.016	81.36758564	0.186
Concentration (nM)	Parental (% intracellular cAMP/total cAMP)	SD
1250	N.A	N.A
250	47.55913148	1.572
50	42.89619279	0.491
10	54.75745033	2.008
2	77.66282246	1.806
0.4	87.65852961	0.039
0.08	90.46191665	0.644
0.016	90.41320801	0.86

Supplementary References

1. Lodowski, D.T., Pitcher, J.A., Capel, W.D., Lefkowitz, R.J. & Tesmer, J.J. Keeping G proteins at bay: a complex between G protein-coupled receptor kinase 2 and Gbetagamma. *Science* **300**, 1256-1262 (2003).
2. Evans, P.R. & Murshudov, G.N. How good are my data and what is the resolution? *Acta crystallographica. Section D, Biological crystallography* **69**, 1204-1214 (2013).
3. Diederichs, K. & Karplus, P.A. Better models by discarding data? *Acta crystallographica. Section D, Biological crystallography* **69**, 1215-1222 (2013).
4. Karplus, P.A. & Diederichs, K. Linking crystallographic model and data quality. *Science* **336**, 1030-1033 (2012).
